# Node, place, ridership, and time model for rail-transit stations: a case study

**DOI:** 10.1038/s41598-022-20209-4

**Published:** 2022-09-27

**Authors:** Ahad Amini Pishro, Qihong Yang, Shiquan Zhang, Mojdeh Amini Pishro, Zhengrui Zhang, Yana Zhao, Victor Postel, Dengshi Huang, WeiYu Li

**Affiliations:** 1 Civil Engineering Department, Sichuan University of Science and Engineering, Zigong, 643000 China; 2 Sichuan University, Chengdu, 610065 China; 3 Southwest Jiaotong University, Chengdu, 610031 China; 4 Université Paris Nanterre, UPL, 92410 Ville d’Avray, France

**Keywords:** Civil engineering, Sustainability, Applied mathematics, Computational science, Scientific data, Statistics

## Abstract

Nowadays, Transit-Oriented Development (TOD) plays a vital role for public transport planners in developing potential city facilities. Knowing the necessity of this concept indicates that TOD effective parameters such as network accessibility (node value) and station-area land use (place value) should be considered in city development projects. To manage the coordination between these two factors, we need to consider ridership and peak and off-peak hours as essential enablers in our investigations. To aim this, we conducted our research on Chengdu rail-transit stations as a case study to propose our Node-Place-Ridership-Time (NPRT) model. We applied the Multiple Linear Regression (MLR) to examine the impacts of node value and place value on ridership. Finally, K-Means and Cube Methods were used to classify the stations based on the NPRT model results. This research indicates that our NPRT model could provide accurate results compared with the previous models to evaluate rail-transit stations.

## Introduction

Public transit operations have now become a logical substitution for private transportation to eliminate the drawbacks such as air pollution and traffic congestion. Transport planners benefit from high-speed trains, subways, and BRTs to implement their cities' Transit-Oriented Development (TOD) concepts. Policymakers, governments, and municipal mayors look forward to providing better access to public transport systems in high-density cities. Thus, comprehensive models for transport planners sound essential. In the past, researchers investigated the potential approaches to match the rail-transit supply and demand. Network accessibility and land use have been considered stem factors to provide the Node-Place (NP) model^1,2^. Researchers define node value based on transport access, network design and structure, and other related network variables. In contrast, they determine place value by assessing the number, diversity, and interaction of urban economic, social, or cultural activities.

The NP model is a regional scaled model concentrating on the rail-transit networks and stations to classify TOD typologies. One of the fundamental ideas of this model is providing the accessibility and conditions for the location to develop the transportation provision. In turn, increasing the demand for transport leads to enhancing the location growth and transport system. The relationship between node value and place value was mentioned in past research^3^. However, it seems there are two more dimensions as necessary as node and place values, which have not been considered yet. Ridership refers to the possibility of using transit centers and infrastructures by public transport takers, which relates to land use and time. To obtain a comprehensive model, we need to collect the data at peak and off-peak hours since the mentioned parameters and ridership are functionally linked with time. Analyzing the coordination between node-place-ridership-time (NPRT) values can better understand this model for transport planners.

As an Asian city and a developing area in China, Chengdu benefits from multiple subway lines, high-speed trains, BRTs, and mono-rails. This brings us an idea to select Chengdu city as our case study to propose a comprehensive model for city planners and municipal government. It sounds beneficial for policymakers to apply an extensive model and know the interaction between NPRT values to provide strategic transit plans. Thus, we aimed to add ridership and time values as third and fourth dimensions to the previous node-place model and proposed a new model to evaluate transit stations.

We structured the remainder of this research to derive our proposed NPRT model and check the accuracy of existing models. In the next section, the previous node-place model and also current research works are reviewed. Section “Methodology and data” covers the methodological approaches and data acquisition. Section “Results and discussion” presents the results, model evaluation, and discussion. Finally, we provide the central conclusion of this research in “Conclusion”.

## Literature review

Peter Calthorpe introduced Transit-Oriented Development (TOD) concept in his book "The Next American Metropolis"^4^. TOD refers to mixed-use and walkable neighborhoods that provide easy access to public transit for people^5^. TOD neighborhoods include transit stations, public centers, high-density residential and commercial buildings, and walkable streets. Classifying station areas based on their similar functional characteristics and set of morphological is the meaning of TOD classification. Distinguishing the types of TOD is a significant concern described in Calthorpe's book^4^. He defined neighborhood TOD and urban TOD according to the spatial orientation of the area functions. Knowing the importance of accessible stations in TOD neighborhoods, many researchers researched how stations can be efficient and reachable.

**Figure 1:**
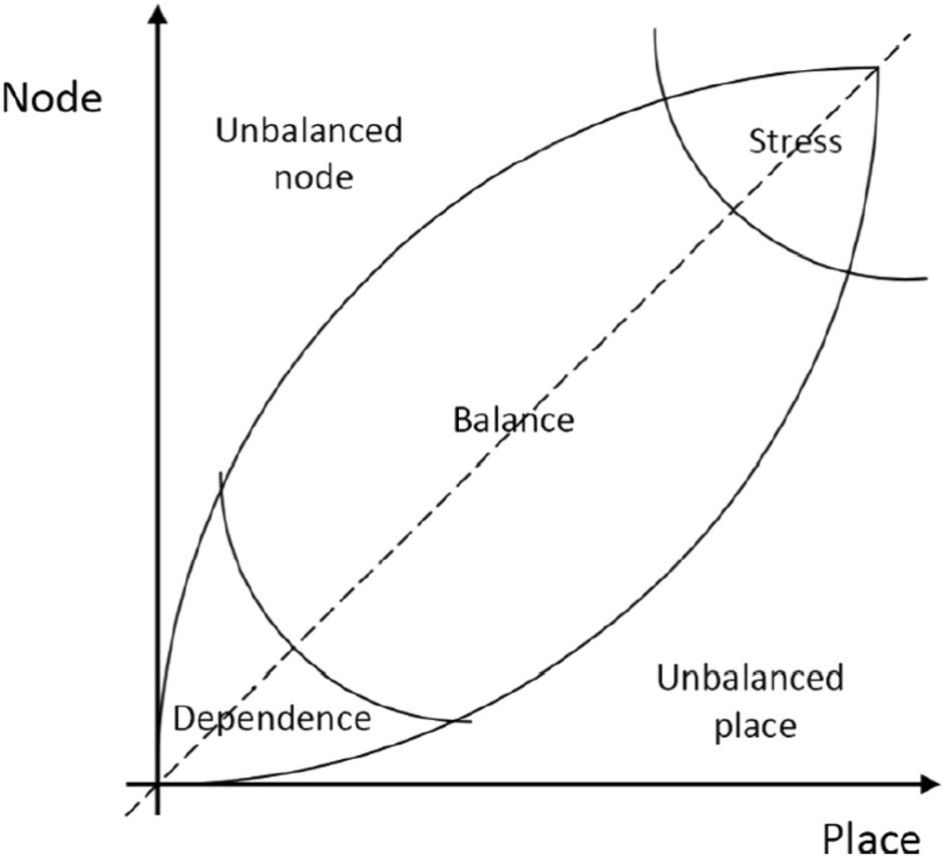
The Node-place model and five ideal–typical situations for a location^1^.

A Node-Place (NP) model was proposed to categorize and evaluate public transit stations in 1999 using node value and place value^1^. As we mentioned before, balancing land use with transportation is the principal aim of the NP model. This model was conveyed in a two-dimensional diagram, as shown in Fig. 1. In this diagram, the station-area land use corresponds to $x$-axis (Place). Place content of an area indicates how human interaction is affected by the diversity of urban activities. Besides, $y$-axis (Node) belongs to the accessibility of the node, which refers to the relationship between people and their interaction. Based on this diagram, five possible situations can be found.

The middle diagonal line area indicates the "Balance" area (1), which means if the node value and place value are similar and equally strong, that station is considered as an accessible or balanced station. In the Balance zone, infra-systems and land use match each other without any stress to maintain the environment and the system. The "Stress" area (2) shows that the diversity of transportation and activities is over-configurated, and the vital node has maximal physical human interaction, making a substantial place value. A station located in the Stress zone has numerous and realized potential facilities to provide a more efficient land use. The "Dependence" area (3) represents stations where the node and place values are matched but under-configuration. In this zone, the demand for public transportation is deficient. There are enough free spaces, but due to the low demand for public transit, there's no reasonable need for infra-system developments. A station is an "Unbalanced node" (4) if transportation facilities are more available than urban activities. In this area, the land use facilities are relatively lower than the public transit flow supply, leading to jammed traffic, massive transit lines, and environmental degradation. A station is considered an "Unbalanced place" (5) if the opposite situation is actual. Land use activities are more available compared to public transportation systems' supply.

A review of the previous research shows that a two-dimensional (node-place) model cannot cover all of the analysis aspects of a station. According to the node-place model, increasing or decreasing the node and/or place value(s) would bring an unbalanced station in the balance area^6,7^. There should be more values to have a comprehensive model since a station with balanced node and place values cannot be efficient or advantageous. In contrast, it does not have a good ridership value. Moreover, the coordination between node, place, and ridership value might not remain steadily constructive in peak and off-peak hours. Therefore, the relationship between node, place, and ridership values should be defined following the time to consider a comprehensive function, including practical values. Engineers and transport planners should design structures and networks concerning the critical situation. Thus, time is as essential as node, place, and ridership values. This research considered all four mentioned values (node, place, ridership, and time) to create our NPRT model to evaluate the efficiency of rail-transit stations.

The node value of a station in the node-place model proposed by Bertolini^1^ is defined as the station's network accessibility, including daily service frequency, the number of stations located in the area within 45 min of traveling, and the number of accessible directions at the station. Other researchers added some indexes to measure the station's node value. Proximity to CBD area by Chorus and Bertolini^8^ and congestion index by Olaru et al.^9^ were added to the node value. At a station, network accessibility includes two significant factors: the accessible opportunities by a station and the transport possibilities to access the opportunities^10–13^. Zhejing Cao et al. recently added accessible opportunities and network centrality into the node value^14^.

Bertolini measured the place value in his proposed node-place model by the station-area land use and the number of residents and employees in economic areas^1^. After Bertolini, other researchers added more indicators to the place value, such as population density, land prices, unemployment rate, number of flats, and core urban area^15,16^. Although density and diversity of activities are primary factors in place value measurement, it seems necessary to consider other essential indicators such as parking areas, fed buses at stations, and walking areas. The built environment features were also included in the place value by Zhejing Cao et al.^14^. Moreover, they studied and considered ridership as the third dimension of node-place value and created the node-place-ridership model.

A comprehensive study on the subway stations and CBDs in Chengdu showed that applying the previous node-place and node-place-ridership models couldn't provide a fair and balanced class for stations. In most case study locations, during weekdays, many people need to change the line, go to work, or come back from their working areas. For example, the subway stations called "South Railway Station" and "Chunxi Road" face a lack of trains and enough space for the riders in the mornings from 6:00 to 9:00 and evenings from 17:00 to 20:00. It's due to the high-frequency trips, in the morning and evening, to and from the working destinations which can be reached via this subway station. Moreover, Chunxi Road is one of the CBDs in Chengdu. There are many shopping malls, offices, consulates, visa centers, and training schools at Chunxi Road station. Let's consider the previous models of node-place and node-place-ridership, in which Time was not considered a leading dimension. It's not possible to justify the reason for these unbalanced stations. These subway stations were designed and categorized without considering time as a significant factor. It can be seen that a station classification might change from balanced to unbalanced several times during the day. A fair comparison and investigation of the existing models proved that it's vital to provide a new and more accurate model for city planners, traffic policymakers, and governments to apply a constructive model to classify the stations based on the needs and demands of the society.

This research work's main contribution and novelty present the Node-Place-Ridership-Time (NPRT) method and the Cube model with 27 classes to provide accurate classifications for rail-transit stations during different time-spans. The NPRT model provides a new contribution to the TOD concept, leading to a more progressive and beneficial policy for cities. To obtain the NPRT model, we added a fourth dimension of Time into the node-place-ridership model to evaluate and classify the transit stations. The coordination between ridership and time influences stations' classification to know which stations are balanced and unbalanced. Without a comprehensive model, we would not establish the relative position of a transit station in the urban regional network. This would assist the city planners and governments in updating their applied policies.

## Methodology and data

### Approach

We consider all of Chengdu city as our case study. First, the Chengdu transit system and the study area are presented in this research. Next, we provide a list of node, place, and ridership indicators. To make our research more accurate, we divide the time into four classes to determine the effect of time on ridership at peak, peak-off, weekend, and other regular hours. Different data resources were used to collect the information for our target variables. We apply the Min–Max Normalization method to normalize our data. Afterward, we apply Information Entropy Weighting (IEW) to combine all indicators and create composite nodes and places. Then, to investigate the relationship between four facets of node, place, ridership, and time, we apply the Multiple Linear Regression (MLR) method. This method investigates the relations between different parameters and factors in scientific research works^17,18^. Afterward, we propose a comprehensive Node-Place-Ridership-Time (NPRT) model. We apply the NP and NPR models proposed by other previous researchers and our proposed NPRT model to evaluate our research work. In this comparison, we use the same database and check all models' accuracy.

### K-Means method

K-Means clustering is one of the most popular unsupervised machine learning algorithms. It is an extensively used technique for data cluster analysis. The goal of this algorithm is to find groups in the data, with the number of groups represented by the variable $K$. The algorithm works iteratively to assign each data point to one of $K$ groups based on the provided features. Data points are clustered based on feature similarity. The steps of K-Means are as follows:

Step 1: Give the parameter $K,$ which means the number of groups we want the points to be assigned to.

Step 2: Randomly select $K$ points as the initial cluster centers ${c}_{1}, {c}_{2},\cdots ,{c}_{K}$.

Step 3: Calculate the distance between each point and each cluster center, then assign it to its nearest center, based on the squared Euclidean distance.

1 $$argmin\,\, dist{({c}_{i}-x)}^{2} 1\le i\le K$$

where $x$ is the point that needs to be assigned to one group.

Step 4: After assigning all the points to the groups, recompute the coordinates of the cluster center, which means replacing the cluster center with the new cluster center.

2 $${c}_{i}=\frac{1}{|{G}_{i}|}\sum_{i=1}^{|{G}_{i}|}{x}_{i}$$

where ${G}_{i}$ means the $i-th$ group, $|{G}_{i}|$ means the number of points in ${G}_{i}$ and ${x}_{i}$ means the $i-th$ point in ${G}_{i}$.

Step 5: Repeat Step3 and Step4 until a stopping criterion is met (i.e., no data points change clusters, the sum of the distances is minimized, or some maximum number of iterations is reached).

**Figure 2:**
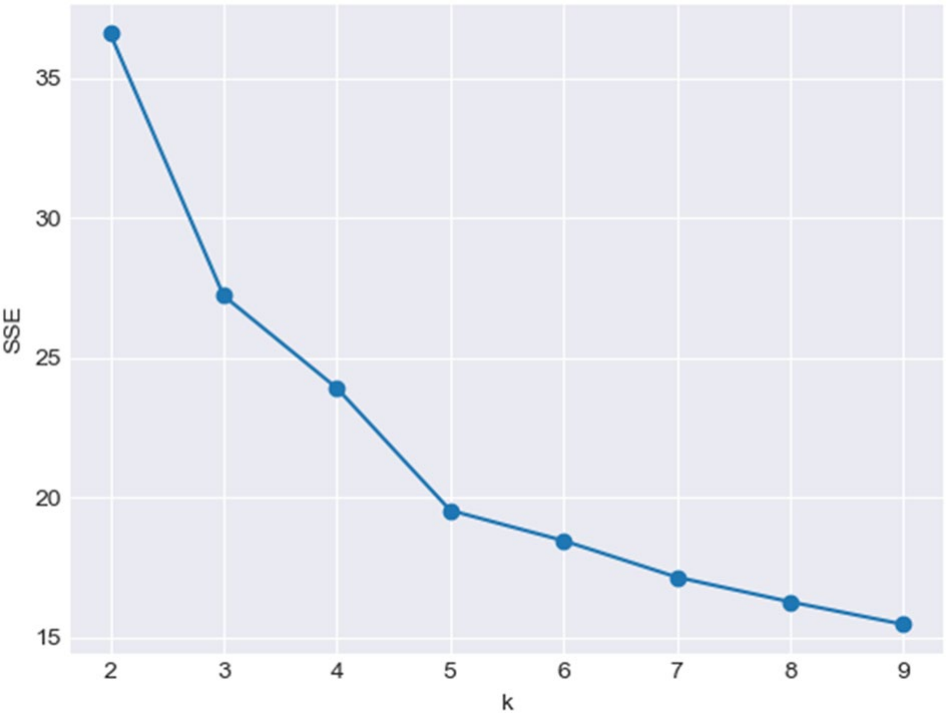
Elbow method and parameter $K$ for the K-Means algorithm.

$K$ value indicates the number of clusters and is a pre-defined value. In this research, we used the Elbow method to select $K$ for the K-Means algorithm^19^. Based on Fig. 2, we can find the value of $K$ where the Sum of Squared Errors $(SSE)$ decreases sharply $(K=5)$.

### Cube method

**Figure 3:**
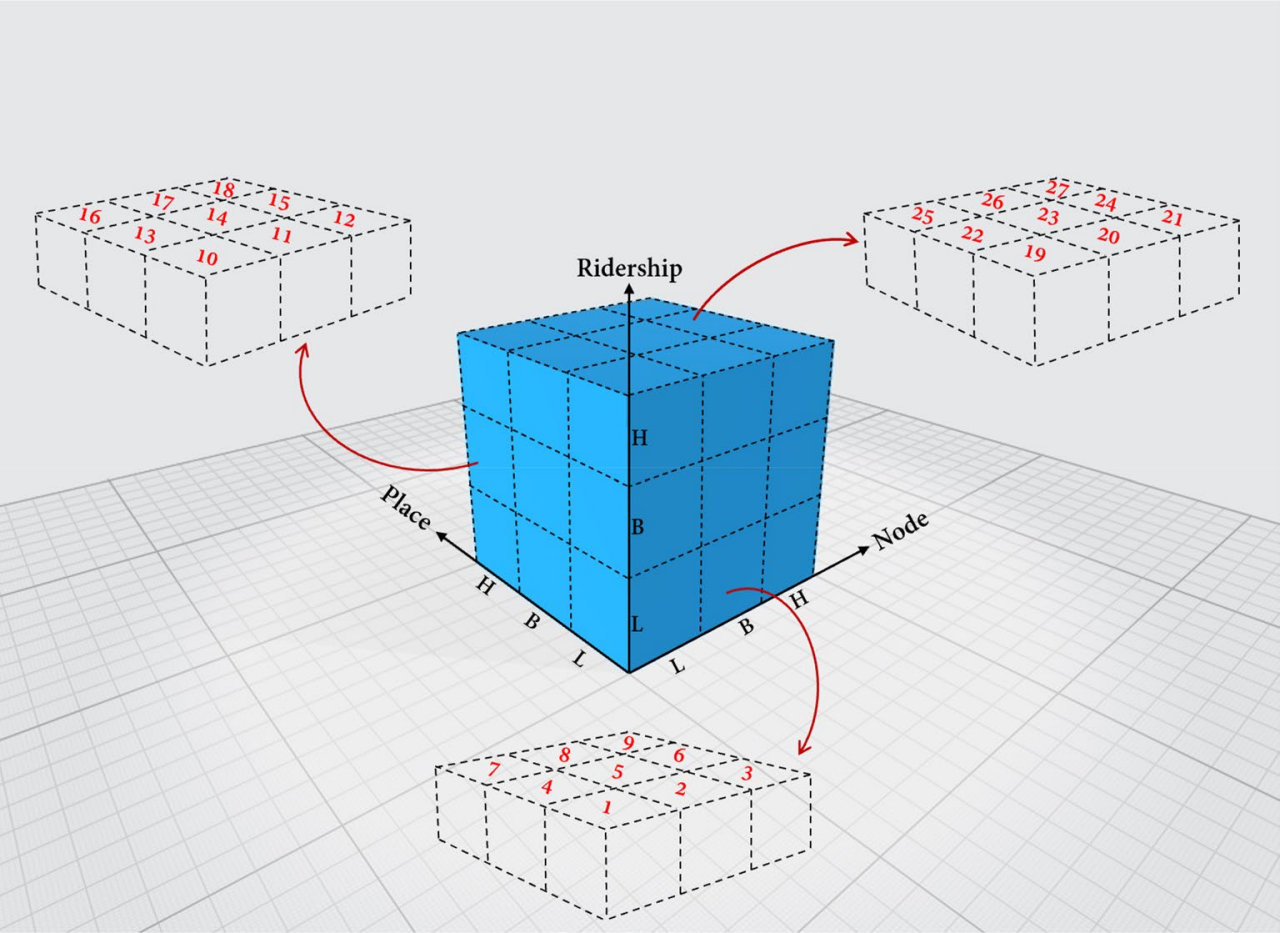
Low Balanced (LB), Balanced (B), and High Balanced (HB) classes of the Cube Method.

To classify our stations, we also applied the Cube method, which is made of 3 dimensions: node value, place value, and ridership regarding the time. According to the Cube method, there are three main layers on each node, place, and ridership measurement value, which are Low Balanced (LB), Balanced (B), and High Balanced (HB), as shown in Fig. 3. The combination of layers on the node, place, and ridership values, leads to 27 classes. Class 1 denotes LB stations in all three values, while class 27 represents HB stations. Cluster 14 means the station is balanced in all three dimensions during the defined time-span. This 3-Dimension illustration provides more understandable coordination between mentioned values and layers compared to the previous models.

Moreover, the Cube method also shows the density of stations in or around critical classes. Therefore, policymakers and city planners can easily understand if their plans need to be revised to improve the efficiency of LB and HB stations. An accurate classification result from 1 to 27 would prove the efficiency of this method, as shown in Appendixes B and C.

To understand the relationship between node, place, ridership, and time values, we applied the K-Means method^19^ using the "sklearn.cluster.KMeans" measure of Python and also the Cube method on NP, NPR, and our proposed NPRT models to classify Chengdu rail-transit stations, shown in Appendixes B to F.

**Figure 4:**
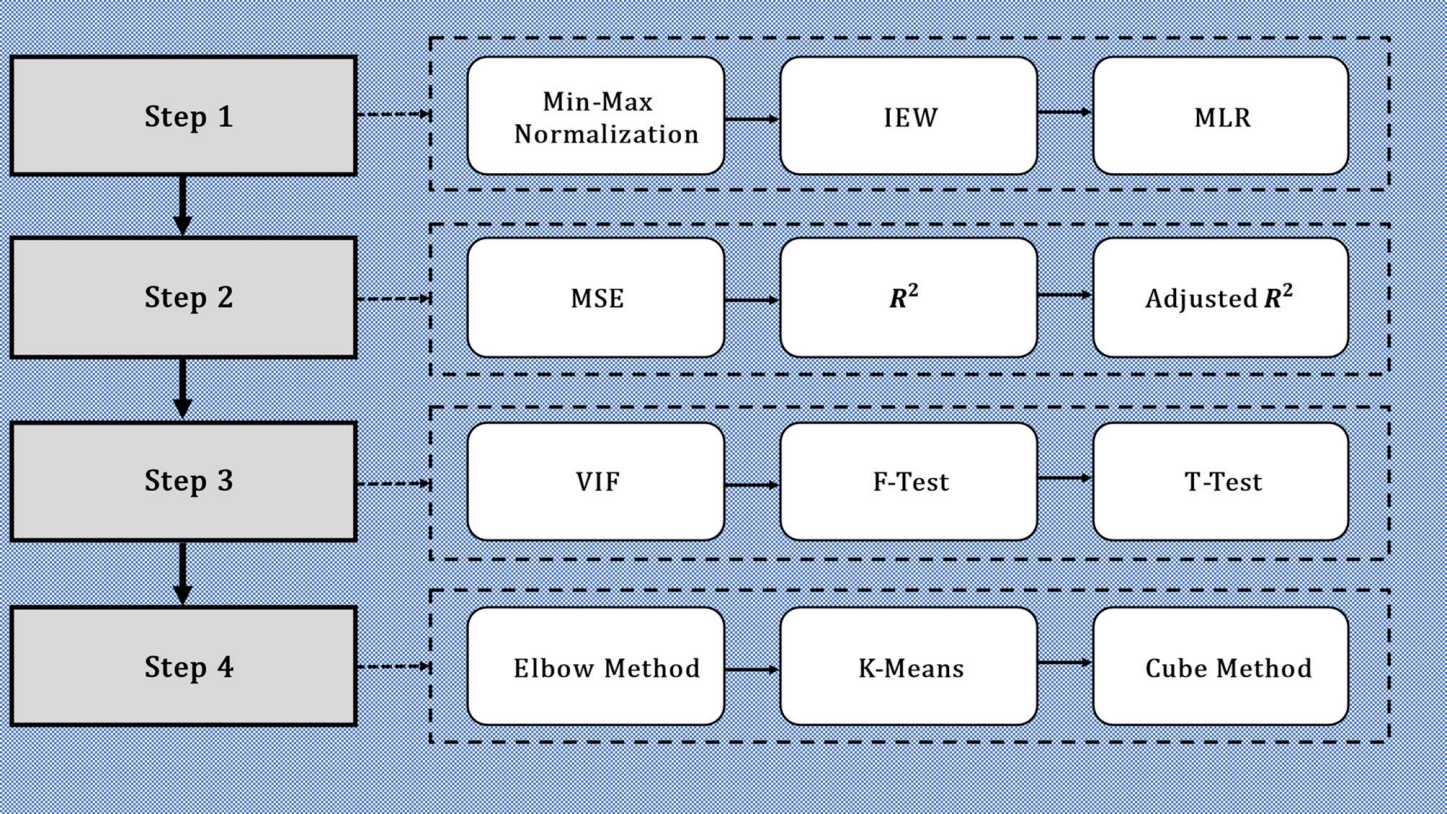
Research structure and applied methods.

As can be extracted from Fig. 4, our research has four main steps. We apply unique methods to prepare, compose, and analyze our data to approach our NPRT model in each step. Table 1 summarizes the application of all the methods used in this research work.

**Table 1:** Description of applied methods.

Method	Application
Min–Max Normalization	For every feature, the minimum value of that feature gets transformed into a 0, the maximum value gets transformed into a 1, and every other value gets transformed into a decimal between 0 and 1
IEW	The Information Entropy Weighting (IEW) is used to combine all indicators and generate a composite node value index and place value index
MLR	Multiple Linear Regression (MLR) is used to model the linear relationship between the ridership and node and place variables
MSE	Mean Squared Error (MSE) is the average squared difference between the estimated and actual values. The MSE is a measure of the quality of an MLR equation
R^2^	In statistics, the coefficient of determination is denoted R^2^ or r^2^. It is pronounced "R squared" is the proportion of the variance in the dependent variable that is predictable from the independent variables R^2^ gives some information about the goodness of fit of an MLR equation
Adjusted R^2^	Adjusted R^2^ is a particular form of R^2^, the coefficient of determination. R^2^ shows how good terms (data points) fit a curve or line. Adjusted R^2^ indicates how well terms fit a curve or line but adjusts for the number of terms in a model
VIF	Variance inflation factor (VIF) measures multicollinearity in multiple regression variables
F-Test	An F-test is any statistical test in which the test statistic has an F-distribution under the null hypothesis. It is most often used when comparing statistical models fitted to a data set to identify the model that best fits the population from which the data were sampled
T-Test	The T-Test is used to judge the significance of each independent variable. If it is significant, the variable significantly impacts the model
Elbow Method	The K-value (number of clusters) is a pre-defined parameter. We Search for the optimal K-value using the Elbow method where the distortion (i.e., within-cluster-sum of squared errors) begins to decrease most rapidly
K-Means	We apply the K-Means method to cluster all stations by their node value, place value, and ridership
Cube	We apply the Cube method to cluster all stations by their node value, place value, and ridership

### The case study area and Chengdu rail transit network

**Figure 5:**
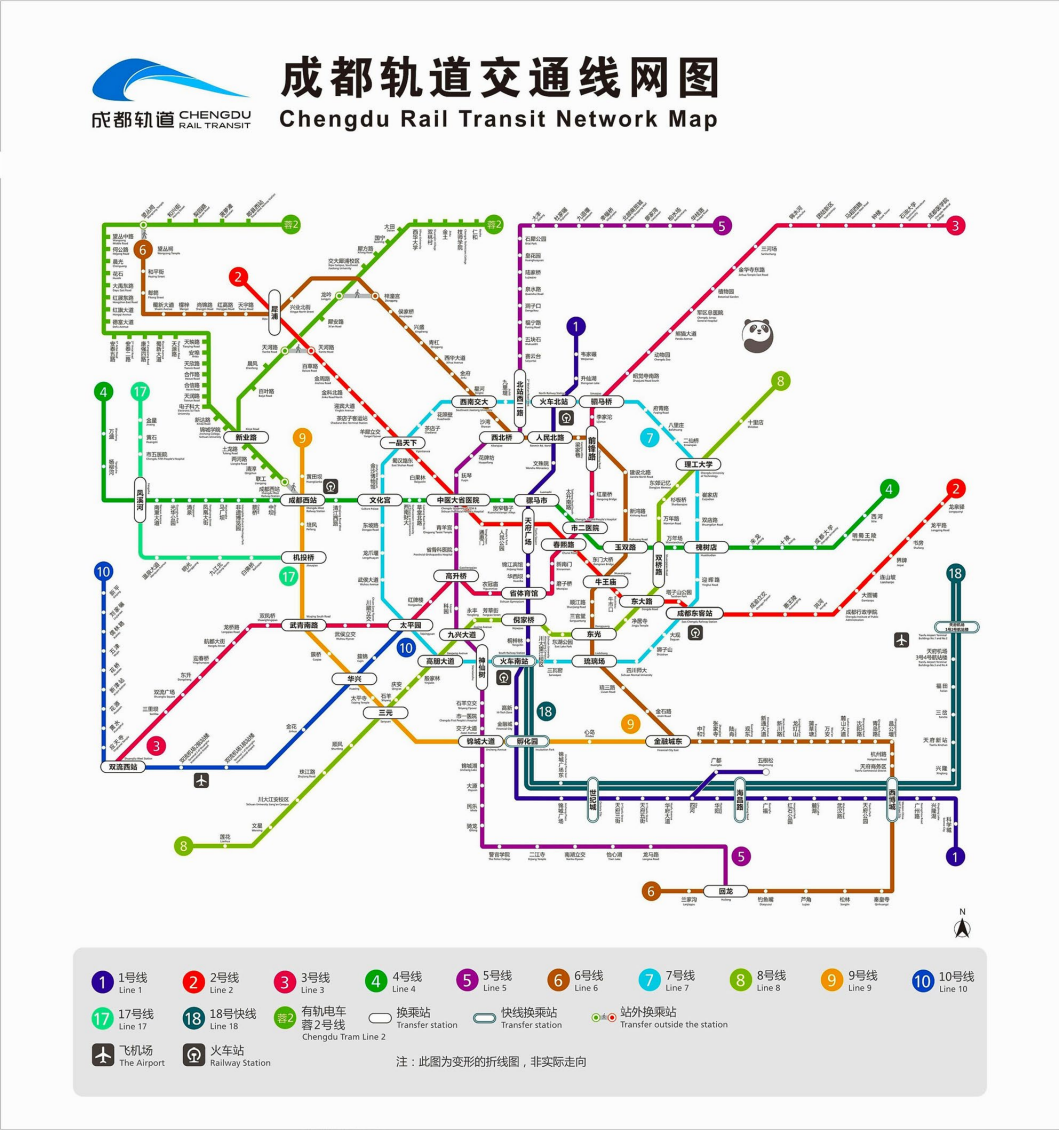
Chengdu rail-transit network and stations.

The Chengdu Metro system is considered the rapid rail-transit network of the capital city of Sichuan province, China, with a daily passenger flow of 5,906,123 rides. The system includes twelve subway lines and one light rail line, operated by Chengdu Rail Transit Group Company. Table 2 presents brief information about Chengdu subway lines. Figure 5 presents the Chengdu rail-transit stations.

**Table 2:** Chengdu metro lines.

Metro Line	Operation date	Newest Extension	Length $(\mathrm{km})$	Stations
1	2010	2018	40.99	35
2	2012	2014	42.32	32
3	2016	2018	49.89	37
4	2015	2017	43.28	30
5	2019	–	49.02	41
6	2020	–	68.88	56
7	2017	–	38.61	31
8	2020	–	29.1	25
9	2020		22.18	13
10	2017	2019	37.972	16
17	2020	–	26.15	9
18	2020	–	69.39	12
Tram R2	2018	2019	39.3	35

### Node, place, ridership, and time indicators

#### Node indicators

We measure a station's node value by four facets: station facility, accessible transits, accessible destinations, and network centrality. Eight node indicators under these four facets are presented in Table 3.

**Table 3:** Node and place indicators.

Dimension	Branch	Indicator	Max	Mean	Min
Node value	Station facility	*N*1. Number of entrances and exits in each metro station (unit)	10.0000	4.6535	2.0000
	Accessible transits	N2. Number of metro stations that one station can reach within 20 min (unit)	88.0000	41.9257	8.0000
		N3. Number of stations to CBD (Chunxi Road) (unit)	23.0000	10.0792	0.0000
		N4. Number of stations to CBD (3rd Tianfu Street) (unit)	33.0000	14.0446	0.0000
	Accessible destinations	N5. Distance to CBD (Chunxi Road) (km)	45.3230	13.6033	0.0000
		N6. Distance to CBD (3rd Tianfu Street) (km)	43.7770	18.3596	0.0000
	Network centrality	N7. Degree centrality	6.0000	2.4653	2.0000
		N8. Closeness centrality (1/1000 km)	0.0004	0.0003	0.0001
Place value	Design	P1. The average price of office land inside the 1000 m—radius catchment area (CNY/m^2^)	74,000.0000	11,118.2658	5550.0000
	Density	P2. Number of offices within 1000 m (unit)	197.0000	26.7673	0.0000
	Design	P3. The average price of commercial land inside the 1000 m − radius catchment area (CNY/m^2^)	50,480.0000	21,285.5855	8571.0000
	Density	P4. Number of shops within 1000 m (unit)	397.0000	117.4554	1.0000
	Design	P5. The average price of residential land inside the 1000 m − radius catchment area (CNY/m^2^)	42,663.3077	18,405.8081	8423.0000
	Density	P6. Number of residences within 1000 m (unit)	552.0000	110.7970	1.0000
	Diversity	P7. Number of public facilities (parks,cultural facilities,schools,hospitals) inside the 1000 m − radius catchment area(unit)	41.0000	10.9208	0.0000
	Design	P8. Number of parking lots inside the 500 m − radius catchment area(unit)	132.0000	21.4851	0.0000
		P9. Number of bus stops inside the 500 m − radius catchment area(unit)	26.0000	7.3515	1.0000

The station facility is measured by the number of entrances and exits (N1) in each metro station. The accessible transits are measured by the number of metro stations (N2) that one station can reach within 20 min, the number of the station to CBD (Chunxi Road) (N3), and the number of stations to CBD (3rd Tianfu Street) (N4). It is well known that there are 2 CBDs in Chengdu: Chunxi Road and 3rd Tianfu Street. Therefore, we calculate the number of stations and the distance to Chunxi Road and 3rd Tianfu Street. The distance measures the accessible destinations to the CBDs Chunxi Road and 3rd Tianfu Street indicated by (N5) and (N6), respectively. The network centrality consists of degree centrality (N7) and closeness centrality (N8).

Based on the graph modeling, we applied the network centrality to capture the impedance of a station in the transit network^20^. To translate the Chengdu rail-transit network into a graph $G = \left( {V,E} \right),$ we assign $V$ of vertices to indicate our stations, and the set $E$ of edges is for the station linkages. The transit traveling distance is used to weigh the $E$^21^. We measure Chengdu network degree centrality (N7) of a transit station $v \in V$ by the number of links connected to station $v$ in Eq. (3), wherein $L_{vt}$ represents the linkage between station $v$ and station $t \in V$, and $K$ shows the number of all stations in set $V$^22^:

3 $$N7\left( v \right) = \mathop \sum \limits_{t = 1}^{K} L_{vt} \left( {v \ne t} \right), L_{vt} = \left\{ {\begin{array}{*{20}c} 1 & {station\;v\; is\; linked\; to\; station\; t} \\ 0 & {station\; v\; is \;not\; linked\;to\;station\;t} \\ \end{array} } \right.$$

Closeness centrality reflects the node's proximity and reachability within the network component. We measure the closeness centrality (N8) of station $v$ by the inverse of the sum of shortest transit distances from station $v$ to all other stations in set $V$ in Eq. (4), wherein $d_{vt}$ denotes the shortest transit distance between station $v$ and station $t \in V$:

4 $$N8\left( v \right) = \frac{1}{{\mathop \sum \nolimits_{t = 1}^{K} d_{vt} }} \left( {v \ne t} \right)$$

Table 4 presents the node indicators values of some stations.

**Table 4:** The values of node indicators in each subway station normalized by Min–Max Normalization method.

Subway station	N1	N2	N3	N4	N5	N6	N7	N8
Weijianian	0.3750	0.4375	0.3043	0.5455	0.1974	0.4479	0.0000	0.6289
Shengxian Lake	0.2500	0.5625	0.2609	0.5152	0.1640	0.4133	0.0000	0.7216
North Railway Station	0.5000	0.8875	0.2174	0.4848	0.1289	0.3769	0.5000	0.8593
Renmin Rd.North	0.6250	0.8250	0.1739	0.4545	0.1028	0.3499	0.5000	0.8778
Wenshu Monastery	0.5000	0.7875	0.1304	0.4242	0.0730	0.3191	0.0000	0.9332
Luomashi	0.3750	1.0000	0.0870	0.3939	0.0535	0.2989	0.5000	0.9769
Tianfu Square	1.0000	0.9750	0.0435	0.3636	0.0311	0.2756	0.5000	1.0000
Jinjiang Hotel	0.2500	0.8250	0.0870	0.3333	0.0494	0.2566	0.0000	0.9720

#### Place indicators

We use a 500-m and 1000-m radius to define the transit catchment area in Chengdu, considering the low-density context of some areas. We measure the station's place value by three facets: design, density, and diversity. Nine place indicators under three facets are presented in Table 3. Table 5 provides the place indicators values of some stations.

**Table 5:** The values of place indicators in each subway station normalized by Min–Max Normalization method.

Subway station	P1	P2	P3	P4	P5	P6	P7	P8	P9
Weijianian	0.0564	0.0254	0.3667	0.1313	0.2113	0.0381	0.1463	0.0076	0.2800
Shengxian Lake	0.0476	0.0152	0.3073	0.2449	0.2000	0.1162	0.2195	0.0379	0.0000
North Railway Station	0.0737	0.1523	0.3318	0.5783	0.2018	0.2976	0.2195	0.2045	0.2800
Renmin Rd.North	0.0747	0.3249	0.3494	0.6162	0.1987	0.4446	0.5854	0.2348	0.2400
Wenshu Monastery	0.0798	0.5533	0.5013	0.5581	0.2449	0.6588	0.3659	1.0000	0.2400
Luomashi	0.0798	0.6650	0.3337	0.6919	0.4187	0.8857	0.5854	0.4242	0.2400
Tianfu Square	0.1032	0.8426	0.3615	0.6970	0.5505	0.6642	0.4634	0.5758	0.3200
Jinjiang Hotel	0.0682	0.5838	0.3828	0.5707	0.6387	0.5245	1.0000	0.5530	0.2800

The design is measured by the average price of office land inside the 1000 m-radius catchment area (P1), the average price of commercial land inside the 1000 m-radius catchment area (P3), the average price of residential land inside the 1000 m-radius catchment area (P5), the number of parking lots inside the 500 m-radius catchment area (P8) and the number of buses stops inside the 500 m-radius catchment area (P9). The design is measured by the number of offices within 1000 m (P2), the number of shops within 1000 m (P4), and the number of residences within 1000 m (P6). The diversity consists of public facilities (parks, cultural facilities, schools, hospitals) inside the 1000 m-radius catchment area (P7).

#### Ridership and time indicators

Because the NPR model's limitation does not consider the implication of time and ignores the difference in ridership about departure and coming, we record the tapped-in and tapped-out arrival trips and construct an NPRT model by considering different conditions.

As we mentioned before, ridership has a direct relationship with time. Therefore, we categorized the passenger traffic into two groups: inbound traffic (I) and the second group for outbound traffic (O). We also divided the time into peak hours, off-peak hours, regular hours, and weekends (T1 to T4). Therefore, we get eight different conditions. IT1 means inbound traffic during working hours, IT2 means inbound traffic during off-hours, IT3 means inbound traffic during the rest of the working day, and IT4 means inbound traffic on two weekend days. OT1 means the ridership of passengers leaving the station during working hours, OT2 means the ridership of passengers leaving the station during off-hours, OT3 means the ridership of passengers leaving the station during the rest of the working day, and OT4 means the ridership of passengers leaving the station on two days of the weekend.

The definition of each class and time-spans from IT1 to OT4 is written in Table 6.

**Table 6:** Time class definition for the NPRT model.

Time	Definition	Days	Hours	Max	Mean	Min
IT1	Inbound traffic during working hours	Monday to Friday	6:00–9:00	27,654.3478	4451.6051	53.2609
IT2	Inbound traffic during off-hours	Monday to Friday	17:00–20:00	46,668.6087	4281.0174	113.6957
IT3	Inbound traffic during the rest of the day	Monday to Friday	9:00–17:00/20:00–23:00	54,702.0870	5156.2546	140.3043
IT4	Inbound traffic on two days of the weekend	Saturdays & Sunday	6:00–23:00	51,955.6250	4607.0829	122.3750
OT1	Passengers leaving the station during working hours	Monday to Friday	6:00–9:00	56,982.3478	5456.5258	151.3913
OT2	Passengers leaving the station during off-hours	Monday to Friday	17:00–20:00	26,532.4783	4367.8530	69.4348
OT3	Passengers leaving the station during the rest of the day	Monday to Friday	9:00–17:00/20:00–23:00	33,976.5217	4064.4983	72.0000
OT4	Passengers leaving the station on both days of the weekend	Saturdays & Sunday	6:00–23:00	55,496.8750	4607.0829	126.6250

Table 7 shows the ridership values of some stations during eight time-spans mentioned above.

**Table 7:** Ridership during different time, normalized by Min–Max Normalization method.

Subway station	IT1	IT2	IT3	IT4	OT1	OT2	OT3	OT4
Weijianian	0.2506	0.0231	0.0409	0.0509	0.0499	0.1558	0.0233	0.0407
Shengxian Lake	0.1132	0.0221	0.0371	0.0323	0.0417	0.0813	0.0343	0.0292
North Railway Station	0.2507	0.1292	0.1828	0.1682	0.2040	0.2335	0.1712	0.1671
Renmin Rd.North	0.1900	0.1732	0.1661	0.1483	0.1550	0.2221	0.2250	0.1387
Wenshu Monastery	0.1748	0.1385	0.1501	0.1166	0.1415	0.1799	0.2142	0.1127
Luomashi	0.1584	0.3132	0.2707	0.1533	0.2191	0.2026	0.5660	0.1559
Tianfu Square	0.1006	0.4388	0.3528	0.2690	0.3215	0.2206	0.6221	0.2636
Jinjiang Hotel	0.0574	0.1339	0.0879	0.0539	0.0742	0.0789	0.2019	0.0534

As for data sources and processing, the number of entrances and exits, offices, shops, residences, parking lots, and bus stops was acquired from Amap (https://www.amap.com/) and SOSO (https://map.qq.com/). The number of stations that one station can reach within 20 min and stations to CBDs (https://www.chengdurail.com/index_en.html), the distance to CBDs, and closeness centrality could be required and calculated via the API of Chengdu Metro Website (https://www.chengdurail.com/index_en.html). The degree of centrality was acquired from a map of Chengdu Metro Station in 2021 in Fig. 5. The average price of office, commercial, and residential land was acquired from Anjuke (https://chengdu.anjuke.com/) and Fang (https://cd.newhouse.fang.com/). We collected ridership of all stations from Chengdu Metro. Each station counts both tapped-in departure trips and tapped-out arrival trips for the station's ridership statistics. All the data was acquired in March 2021.

### Information entropy weighting (IEW)

To practice our data analysis and compose the indicators, we applied Information Entropy Weighting (IEW)^23^ to provide a composite node or place value index. We use the IEW method to integrate $N1 - N8$ into one Node value and $P1 - P9$ into one Place value.

First, the decision matrix should be constructed, shown in Eq. (5). $m$ stations and $n$ node value indicators have consisted in $X$. Moreover, $X_{pq}$ indicates the value of indicator $q$ at station $p$. We apply Eq. (6) to normalize the decision matrix:

5 $$X = \left\{ {X_{pq} } \right\}_{m \times n}$$

6 $$X_{pq}^{^{\prime}} = \frac{{X_{pq} - \min \left\{ {X_{q} } \right\}}}{{\max \left\{ {X_{q} } \right\} - \min \left\{ {X_{q} } \right\}}}$$

Then, $R_{pq}^{^{\prime}}$ computes the proportion of station $p$ for indicator $q$:

7 $$R_{pq}^{^{\prime}} = \frac{{X_{pq}^{^{\prime}} }}{{\mathop \sum \nolimits_{p = 1}^{m} X_{pq}^{^{\prime}} }}$$

We can calculate the entropy value $e_{q}$ of indicator $q$ in Eq. (8), knowing that if $R_{pq}^{^{\prime}} = 0$, then $\ln R_{pq}^{^{\prime}} = 0.$

8 $$e_{q} = - \frac{1}{\ln m} \times \mathop \sum \limits_{p = 1}^{m} R_{pq}^{^{\prime}} \cdot \ln R_{pq}^{^{\prime}}$$

In the next step, we need to calculate the imbalance coefficient using Eq. (9):

9 $$g_{q} = 1 - e_{q}$$

$W_{q}$ is the weight of indicator $q$, which can be extracted from Eq. (10). Then, to compose the node value index $N_{p}$ for station $p$, we can apply Eq. (11):

10 $$W_{q} = \frac{{g_{q} }}{{\mathop \sum \nolimits_{q = 1}^{n} g_{q} }}$$

11 $$N_{p} = \mathop \sum \limits_{q = 1}^{n} W_{q} \times X_{pq}^{^{\prime}}$$

Afterward, we need to normalize the node value index between 0 and 1. In Eq. (12), $N$ indicates the array of node value index, $m$ is the number of stations, and $p$ is the target station:

12 $$N_{p}^{^{\prime}} = \frac{{N_{p} - \min \left\{ N \right\}}}{{\max \left\{ N \right\} - \min \left\{ N \right\}}}$$

## Results and discussion

### Equations

We obtain the equations through Multiple Linear Regression (MLR). Table. 8 provides a list of constants and variables coefficients of our equations. The results of our MLR models are presented in Table 9.

**Table 8:** Constants and variable coefficients of MLR models.

Coefficient	MLR models							
	IT1	IT2	IT3	IT4	OT1	OT2	OT3	OT4
α	0.5098	0.1951	0.2225	0.2439	0.2318	0.4979	0.1893	0.2196
β_1_	− 0.0409	− 0.0596	− 0.0915	− 0.0747	− 0.0809	− 0.0804	− 0.0572	− 0.0722
β_2_	− 0.223	0.0848	− 0.005	− 0.0224	− 0.0312	− 0.081	0.0797	− 0.0076
β_3_	0.169	0.0997	0.0111	0.0109	0.0087	0.1586	0.1433	0.0077
β_4_	− 0.3049	− 0.1247	− 0.1031	− 0.1239	− 0.1118	− 0.304	− 0.1265	− 0.1063
β_5_	− 0.6797	− 0.3089	− 0.2937	− 0.3087	− 0.3025	− 0.7084	− 0.315	− 0.281
β_6_	0.0204	0.0581	0.087	0.0893	0.0874	0.0922	0.0217	0.0831
β_7_	0.1211	0.1132	0.1715	0.1486	0.1571	0.1573	0.1334	0.141
β_8_	− 0.2113	− 0.2221	− 0.1939	− 0.2033	− 0.1928	− 0.3575	− 0.184	− 0.2047
γ_1_	0.0825	− 0.0686	− 0.0762	− 0.0621	− 0.0591	0.0345	− 0.086	− 0.0592
γ_2_	− 0.2814	0.4859	0.2112	0.1221	0.1617	− 0.0831	0.6999	0.1378
γ_3_	0.0568	0.0161	0.0242	0.0254	0.0356	0.073	0.0187	0.032
γ_4_	0.4188	0.1068	0.1719	0.2367	0.2271	0.4613	0.017	0.2269
γ_5_	0.1175	0.0382	0.0354	0.0244	0.0272	− 0.0449	0.0201	0.0275
γ_6_	− 0.1733	− 0.1741	− 0.1119	− 0.1041	− 0.1129	− 0.1663	− 0.2469	− 0.1006
γ_7_	0.0711	− 0.0558	− 0.0085	− 0.028	− 0.0204	0.0016	− 0.0084	− 0.0298
γ_8_	0.1597	0.0447	0.0686	0.0485	0.0642	0.1113	0.0908	0.0427
γ_9_	0.1273	0.0234	0.0305	0.0196	0.0311	0.1049	0.0523	0.0187

**Table 9:** MLR models results.

	IT1	IT2	IT3	IT4	OT1	OT2	OT3	OT4
Adjusted R^2^	0.3875	0.5946	0.3221	0.2689	0.3284	0.3499	0.6981	0.2983
R^2^	0.4393	0.6289	0.3794	0.3307	0.3852	0.4049	0.7236	0.3576
MSE	0.0090	0.0053	0.0081	0.0068	0.0067	0.011	0.0065	0.0059
F-Test	8.4800 ****	18.339 ****	6.6173 ****	5.3487 ****	6.7814 ****	7.3642 ****	28.34 ****	6.0256 ****
T-Constant	4.2463 ****	2.122 **	1.9528 *	2.3366 **	2.2334 **	3.7494 ****	1.8495 *	2.2587 **
T – N1	− 0.9934	− 1.8903 *	− 2.3418 **	− 2.0869 **	− 2.2731 **	− 1.7656 *	− 1.6297	− 2.1656 **
T – N2	− 1.6851 *	0.8367	− 0.0398	− 0.1947	− 0.2727	− 0.5534	0.7064	− 0.0709
T – N3	1.5681	1.2079	0.1085	0.1163	0.0934	1.3304	1.5596	0.0882
T − N4	− 3.0495 ***	− 1.6286	− 1.0865	− 1.4253	− 1.2935	− 2.7489 ***	− 1.4841	− 1.3129
T – N5	− 3.6314 ****	− 2.155 **	− 1.6533 *	− 1.8969 *	− 1.8695 *	− 3.4217 ****	− 1.974 **	− 1.8538 *
T – N6	0.1772	0.659	0.7963	0.8922	0.8782	0.7241	0.2211	0.8913
T – N7	2.7893 ***	3.4046 ****	4.1622 ****	3.9367 ****	4.1857 ****	3.2756 ****	3.6041 ****	4.0104 ****
T – N8	− 1.0942	− 1.5018	− 1.058	− 1.2109	− 1.1549	− 1.6737 *	− 1.1177	− 1.309
T – P1	0.9914	− 1.0764	− 0.9648	− 0.8583	− 0.8215	0.3748	− 1.2122	− 0.8785
T – P2	− 3.9816 ****	8.9774 ****	3.1487 ***	1.9871 **	2.6466 ***	− 1.063	11.616 ****	2.4077 **
T – P3	0.7343	0.2718	0.3297	0.3777	0.5324	0.8533	0.2836	0.5109
T – P4	4.7953 ****	1.5968	2.0739 **	3.1172 ***	3.0079 ***	4.7753 ****	0.2283	3.2082 ***
T – P5	− 2.0838 **	0.8846	0.6615	0.4977	0.558	− 0.7199	0.4181	0.6022
T – P6	− 1.9438 *	− 2.5499 **	− 1.3225	− 1.343	− 1.4648	− 1.6863 *	− 3.2483 ****	− 1.3933
T – P7	1.0671	− 1.0935	− 0.1344	− 0.4833	− 0.3542	0.0217	− 0.1479	− 0.5523
T – P8	1.7577 *	0.6424	0.7956	0.614	0.8174	1.1075	1.1723	0.5803
T – P9	2.4117 **	0.5789	0.6088	0.4271	0.6815	1.7967 *	1.1622	0.4375

Variance Inflation Factor (VIF) = 10.7532.

If p value < 0.001 ⇒ ****; p value < 0.01 ⇒ ***; p value < 0.05 ⇒ **; p value < 0.1 ⇒ *.

The general format of our MLR equations is as follows:

13 $$Ridership = \alpha + \mathop \sum \limits_{i = 1}^{8} \beta_{i} N_{i} + \mathop \sum \limits_{j = 1}^{9} \gamma_{j} P_{j}$$

where $\alpha$ is the equation constant, and $\beta$ and $\gamma$ are the coefficient of node value and place value, respectively.

To better understand how a station's node value and place value impact its ridership at different times, we must analyze our eight MLR models below. Concerning the parameters of eight MLR models, we can know that the number of entrances and exits, the number of stations to CBD (3rd Tianfu Street), the distance to CBD (Chunxi Road), closeness centrality, and the number of residences within 1000 m are negatively associated with ridership in all facets of time. The number of stations to CBD (Chunxi Road), the distance to CBD (3rd Tianfu Street), degree of centrality, the average price of commercial land, the number of shops within 1000 m, and the number of parking lots and bus stops inside the 500 m-radius catchment area are positively associated with ridership in all facets of time.

The number of stations that one station can reach within 20 min is positively associated with ridership of stations in off-peak hours, is positively associated with ridership of getting outstations in other hours on working days, and is negatively associated with ridership in other times. The average price of office land and the number of public facilities are positively associated with ridership of getting in stations in peak hours, are positively associated with ridership of getting outstations in off-peak hours, and are negatively associated with ridership in other times. The number of offices within 1000 m and the average price of residential land are negatively associated with ridership of getting in stations in peak hours, are negatively associated with ridership of getting outstations in off-peak hours, and are positively associated with ridership in other times.

The distance to CBD (Chunxi Road) is significantly negatively associated with ridership of getting in stations in peak hours and ridership of getting outstations in off-peak hours. The number of offices within 1000 m is significantly positively associated with ridership of getting in stations in off-peak hours and ridership of getting outstations on other working days. The number of shops is incredibly positively associated with the ridership of getting in peak hours and the ridership of getting outstations in off-peak hours.

Using Table 8 in the MLR Eq. (13), we have eight equations from ${\text{IT}}1$ to ${\text{OT}}4.$ For instance, the equation of Inbound traffic during working hours from 6:00 a.m. to 9:00 a.m. would be as follows:

14 $$IT1 = 0.5098 - 0.0409N1 - 0.223N2 + 0.169N3 - 0.3049N4 - 0.6797N5 + 0.0204N6 + 0.1211N7 - 0.2113N8 + 0.0825P1 - 0.2814P2 + 0.0568P3 + 0.4188P4 - 0.1175P5 - 0.1733P6 + 0.0711P7 + 0.1597P8 + 0.1273P9$$

All variables have been 0–1 normalized by Min–Max Normalization for the model input, shown in Appendix A. The variance inflation factor (VIF) is approximately equal to 10, indicating no severe multicollinearity. The adjusted R^2^ and R^2^ are more extensive than 0.25, showing that the results are promising in model fitting. When using 0.05 as a significance level threshold, F-test shows that our MLR models are significant. The T-Test shows the number of stations to CBD (3^rd^ Tianfu Street), the distance to CBD (Chunxi Road), degree of centrality, the number of offices within 1000 m, the number of shops within 1000 m, the average price of residential land and the number of bus stops are significant with equation IT1. The distance to CBD (Chunxi Road), degree of centrality, the number of offices, and the number of residences are significant with equation IT2. The number of entrances and exits, degree of centrality, the number of offices, and the number of shops are significant with equations IT3, IT4, and OT1. The number of stations to CBD (3^rd^ Tianfu Street), the distance to CBD (Chunxi Road), degree centrality, and the number of shops are significant with equation OT2. The distance to CBD (3^rd^ Tianfu Street), the number of offices, and the number of residences are significant with equation OT3. The number of entrances and exits, degree of centrality, the number of offices, and the number of shops are significant with equation OT4.

### Methods and classification results

The coordination between ridership and time influences stations' classification to know which stations are balanced and unbalanced.

**Figure 6:**
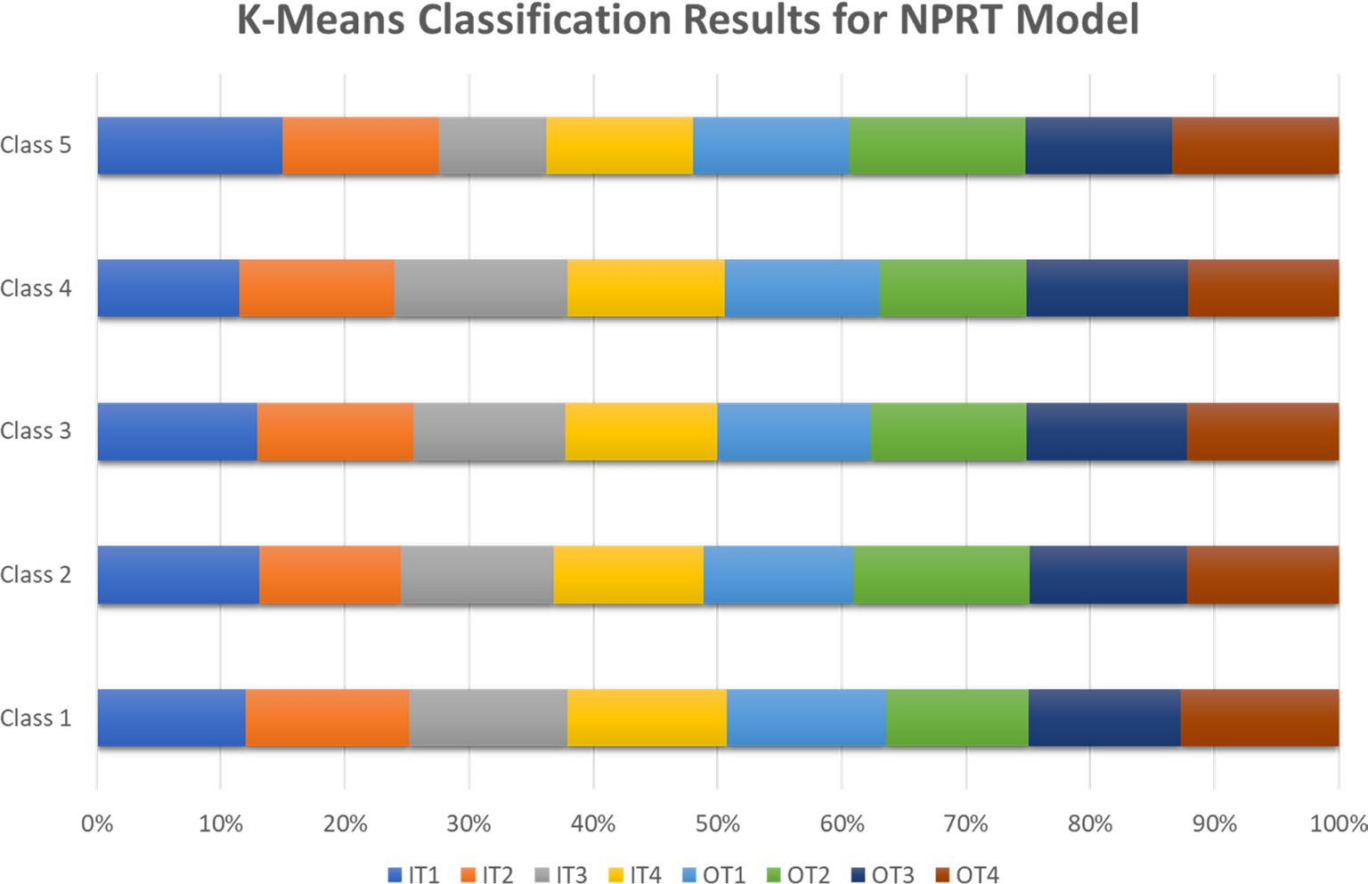
Number of stations in K-Means classification method for NPRT model.

Regarding the node value, place value, and ridership extracted in four time-spans, five classes resulted from the K-Means method. Figure 6 summarizes the classification results extracted from the K-Means method for our proposed NPRT model.

In each model from IT1 to OT4, shown in Appendix B, F, and Fig. 6, based on the NPRT values, the results show that some stations can be balanced or unbalanced; low, medium, high, or extremely high ridership; stress or dependent.

For example, in the model IT1, Xipu station with the result of [0.4523, 0.2703, 1.0] for the node value, place value, and ridership is categorized in class 4, with high ridership and balanced, while Chunxi Road station with the values of [0.5185, 0.8886, 0.1691], is in class 5, low ridership and unbalanced place. Compared to the IT4 model, on weekends, Xipu station is medium ridership and balanced class 4, with the NPR values of [0.4523, 0.2703, 0.4807]. Chunxi Road station for the same model indicates the results of [0.5185, 0.8886, 1.0], falling into class 5, extremely high ridership, and an unbalanced place. Therefore, it can be seen that although the node and place values are essential factors in our classifications model, the ridership at different time-spans can significantly change the results.

**Figure 7:**
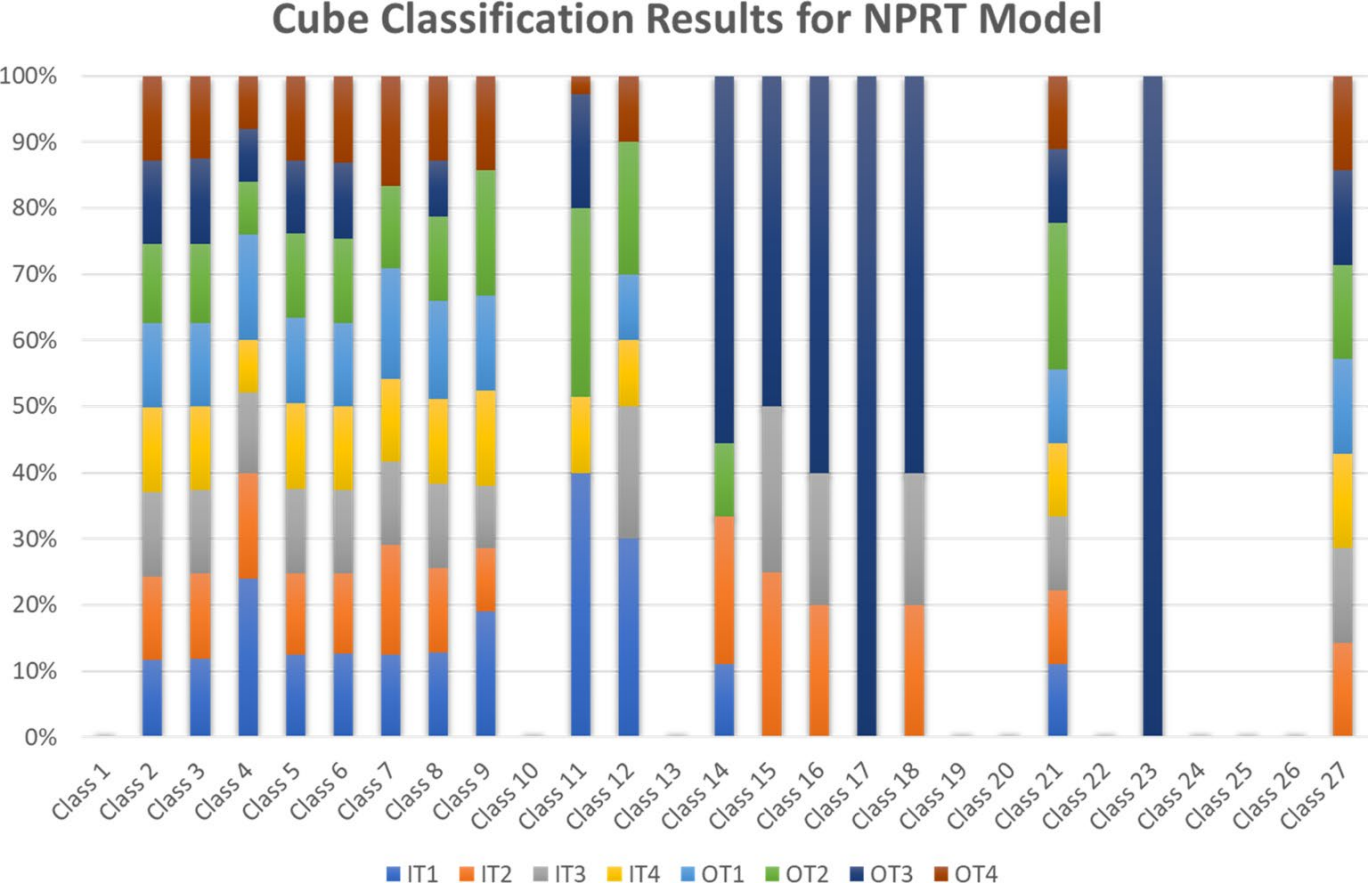
Number of stations in Cube classification method for NPRT model.

As already mentioned, in both K-Means and Cube methods, the concept of ridership is influenced by time. The relationship between ridership and time can also be proved by analyzing the results of the Cube Method. The number of stations in each class of the Cube method is presented in Fig. 7. Based on Fig. 3, class 1 has a low node, place, and ridership values, while class 27 comprises high node, place, and ridership values.

Regarding Appendix C, F, and Fig. 7, class 2 includes 52.8% to 58.04% of Chengdu rail-transit stations. In contrast, some other classes, such as class 1, have 0% of the stations. This difference is not only because of the low or high node and place value, but the time as a significant factor in ridership caused the differences as mentioned. Some stations have good status on node and place values. If we only consider the ridership value as a constant value during our investigation, the results would be different from the reality. Stations can be balanced at 1 h but unbalanced at another hour. As an instance, in the OT1 model, Chunxi Road, for NPR values of [0.5185, 0.8886, 1.0] is classified in cluster 27, high node, place, and ridership value, whereas this station in the IT1 model is in class 9, [0.5185, 0.8886, 0.1691], high node and place value, but low ridership. Chunxi Road is one of the CBDs of Chengdu. It is surrounded by many shopping malls, companies, institutions, consulates and visa centers, and headquarters. During the time-span OT1, 6:00–9:00 a.m. on weekdays (Table 6), the number of passengers going to work in the Chunxi Road area is considerably higher than the number of people traveling from this location to the other part of the city (IT1 model). Therefore, we can find the influence of time on the ridership and, consequently, on the classification of a station.

To compare our proposed NPRT model with the NP model by Bertolini^1^ and the NPR model by Zhejing Cao^14^, we also applied our case study area and rail-transit stations to the NP and NPR models, presented in Appendixes D to F.

Regarding many subway stations in Chengdu and the massive number of classification results, we provided the results of four stations in Table 10 as a sample. Table 10 shows the station classifications resulting from the K-Means method and Cube model, using NP, NPR, and NPRT. The results prove that Chunxi Road was classified as an unbalanced station with high NPR values over different time-spans, while the Financial City is a balanced station during OT3 (class 14 of the Cube model). According to our previous discussion about Fig. 2, there are five classes in the K-Means method (K = 5). In comparison, the Cube model provides 27 classes, leading to more accurate classifications. For instance, in Table 10, the K-Means method for the Chunxi Road station has the same result (class 5) during the time-spans IT1 and IT2, whereas the Cube method puts this station at class 9 during IT1 and class 27 over IT2. The results of the Xipu station experience the same situation for the time-spans IT1, IT2, OT3, and OT4.

**Table 10:** Stations classification results (NP, NPR, NPRT).

Station	Method	Node value (N)	Place value (P)	Ridership value (R)	Time span (T)	Class type
Chunxi Road	NP	0.5185	0.8886	–	–	4
	NPR [K–Means]	0.5185	0.8886	1.0	–	5
	NPRT [K-Means]	0.5185	0.8886	0.1691	IT1	5
	NPRT [Cube]					9
	NPRT [K-Means]	0.5185	0.8886	1.0	IT2	5
	NPRT [Cube]					27
	NPRT [K-Means]	0.5185	0.8886	1.0	IT3	5
	NPRT [Cube]					27
	NPRT [K-Means]	0.5185	0.8886	1.0	IT4	5
	NPRT [Cube]					27
	NPRT [K-Means]	0.5185	0.8886	1.0	OT1	5
	NPRT [Cube]					27
	NPRT [K-Means]	0.5185	0.8886	1.0	OT2	5
	NPRT [Cube]					27
	NPRT [K-Means]	0.5185	0.8886	0.853	OT3	5
	NPRT [Cube]					27
	NPRT [K-Means]	0.5185	0.8886	1.0	OT4	5
	NPRT [Cube]					27
Financial City	NP	0.0605	0.4265	–	–	4
	NPR [K-Means]	0.0605	0.4265	0.2096	–	2
	NPRT [K-Means]	0.0605	0.4265	0.054	IT1	3
	NPRT [Cube]					5
	NPRT [K-Means]	0.0605	0.4265	0.3215	IT2	3
	NPRT [Cube]					5
	NPRT [K-Means]	0.0605	0.4265	0.1412	IT3	2
	NPRT [Cube]					5
	NPRT [K-Means]	0.0605	0.4265	0.0671	IT4	2
	NPRT [Cube]					5
	NPRT [K-Means]	0.0605	0.4265	0.1212	OT1	2
	NPRT [Cube]					5
	NPRT [K-Means]	0.0605	0.4265	0.0852	OT2	3
	NPRT [Cube]					5
	NPRT [K-Means]	0.0605	0.4265	0.5194	OT3	3
	NPRT [Cube]					14
	NPRT [K-Means]	0.0605	0.4265	0.0747	OT4	2
	NPRT [Cube]					5
Southwest Jiaotong University	NP	0.5718	0.4296	–	–	2
	NPR [K-Means]	0.5718	0.4296	0.0828	–	4
	NPRT [K-Means]	0.5718	0.4296	0.089	IT1	4
	NPRT [Cube]					6
	NPRT [K-Means]	0.5718	0.4296	0.0678	IT2	4
	NPRT [Cube]					6
	NPRT [K-Means]	0.5718	0.4296	0.0658	IT3	4
	NPRT [Cube]					6
	NPRT [K-Means]	0.5718	0.4296	0.0533	IT4	4
	NPRT [Cube]					6
	NPRT [K-Means]	0.5718	0.4296	0.0669	OT1	4
	NPRT [Cube]					6
	NPRT [K-Means]	0.5718	0.4296	0.0982	OT2	4
	NPRT [Cube]					6
	NPRT [K-Means]	0.5718	0.4296	0.1058	OT3	4
	NPRT [Cube]					6
	NPRT [K-Means]	0.5718	0.4296	0.0555	OT4	4
	NPRT [Cube]					6
Xipu	NP	0.4523	0.2703	–	–	2
	NPR [K-Means]	0.4523	0.2703	0.5461	–	4
	NPRT [K-Means]	0.4523	0.2703	1.0	IT1	4
	NPRT [Cube]					21
	NPRT [K-Means]	0.4523	0.2703	0.2357	IT2	4
	NPRT [Cube]					3
	NPRT [K-Means]	0.4523	0.2703	0.4422	IT3	4
	NPRT [Cube]					12
	NPRT [K-Means]	0.4523	0.2703	0.4807	IT4	4
	NPRT [Cube]					12
	NPRT [K-Means]	0.4523	0.2703	0.4997	OT1	4
	NPRT [Cube]					12
	NPRT [K-Means]	0.4523	0.2703	0.8806	OT2	5
	NPRT [Cube]					21
	NPRT [K-Means]	0.4523	0.2703	0.2356	OT3	4
	NPRT [Cube]					3
	NPRT [K-Means]	0.4523	0.2703	0.4351	OT4	4
	NPRT [Cube]					12

Moreover, the results show that the classification result to check the station efficiency would not be accurate without considering the relationship between ridership value and time. For example, comparing Chunxi Road station in three different models, we can see the node value, place value, and the ridership in NP, NPR, and NPRT IT1 models are [0.5185, 0.8886, –-], [0.5185, 0.8886, 1.0], and [0.5185, 0.8886, 0.1691], respectively. This station's ridership for the NPR model is extremely high, although it is low for the NPRT IT1 model. This situation is true for some other stations, such as North Railway Station, Wenshu Monastery, Tianfu Square, Sichuan Gymnasium, Hi-Tech Zone, Financial City, and Century City. Therefore, as one of the most important factors in policymaking, the ridership should be considered regarding the critical time-spans, from IT1 to OT4.

The periods IT1 to OT4, NPRT method, and Cube model can assist the policymakers and city planners update their applied policies. We can consider the Chunxi Road station as an example. The NPR values at the time-span OT1, [0.5185, 0.8886, 1.0], and its class 27 would let the municipal government know this location needs some charter trains at the time-span OT1. The charter trains would travel directly between the high frequently-demanded stations to the Chunxi Road. The Chengdu Metro Co. can calculate the frequency and number of required charter trains by knowing the number of riders during the critical time-span.

Moreover, since the Chunxi Road station is located at the junction of lines 2 and 3, the Chengdu Metro Co. would be able to find the Low Balanced (LB) stations on lines 2 and 3 at the time-spans T1. Therefore, every second train can stop at the LB stations during the time-span T1. Southwest Jiaotong University is almost a steady station (class 6). Since this station is located in the Jinniu district, the Jinniu municipal government would be able to apply the results of this study in their potential plans to enhance the classification of this station toward cluster 14, which creates a fully balanced station.

These are some examples of potential revised policies based on this study's innovation in developing the station classifications. Regarding the 27 classes from the Cube model and the NPRT method, governments can access the accurate classification results of the stations during critical time-spans T1 to T4 to implement appropriate policies and enhance the rail-transit network efficiency.

## Conclusion

In this research, we conducted a case study on Chengdu rail-transit stations to present the relationship between node, place, and ridership. Since the number of riders during the daytime and over the week is not constant, we divided our investigation into four time-spans. It was proved that ridership has a direct relationship with time. So, we included this factor in our study. After collecting the data and providing all the influential parameters, Multiple Linear Regression (MLR) was applied to create our Node-Place-Ridership-Time (NPRT) equations. MLR is a constructive method to model the coordination and relationship between the effective parameters on the NPRT model. We developed our classifications using k-Means and Cube methods and analyzed the results. Stations with exemplary node and place values can not be necessarily balanced or efficient since the ridership and time-span play essential roles on the other side. The policymakers, city planners, and governments need to apply NPRT models to analyze the efficiency of transit stations. Compared with Node-Place (NP) and Node-Place-Ridership (NPR) models presented by previous researchers, our proposed NPRT model provides more accurate results.

### Possible directions for future studies

This research investigated the impact of node, place, and time values on ridership to present the NPRT model for classifying rail-transit stations. However, the effect of ridership on node and place values which leads to the bi-directional relationship between the dependent and independent variables would be an open discussion for future studies. Moreover, the effect of the economy, ecology, and sociodemographic characteristics (such as transit mode share, household going-out rate, and age composition) on the NPRT model would be essential for future studies.

## Supplementary Information

Supplementary Information.

## Data Availability

The datasets used and/or analysed during the current study available from the corresponding author on reasonable request.
