# Supplementary material for: Node, place, ridership, and time model for rail-transit stations: a case study
Source: Sci Rep. 2022 Sep 27;12:16120. doi: 10.1038/s41598-022-20209-4 (PMC9515214; doi:10.1038/s41598-022-20209-4)
Supplement: Supplementary file 1 — Supplementary Information. [file 41598_2022_20209_MOESM1_ESM.docx]

**Appendix A. Normalization**


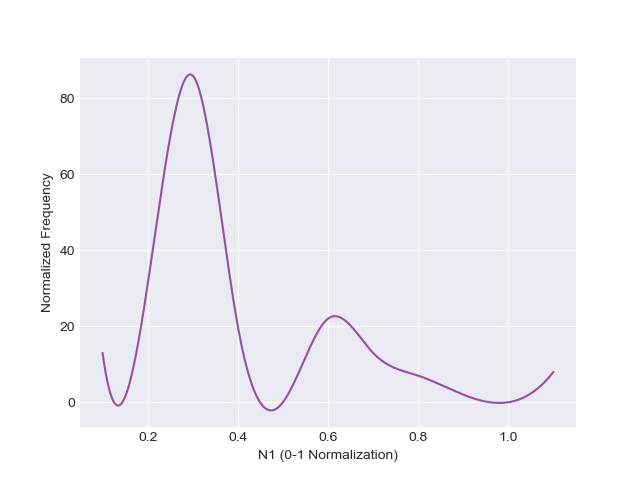

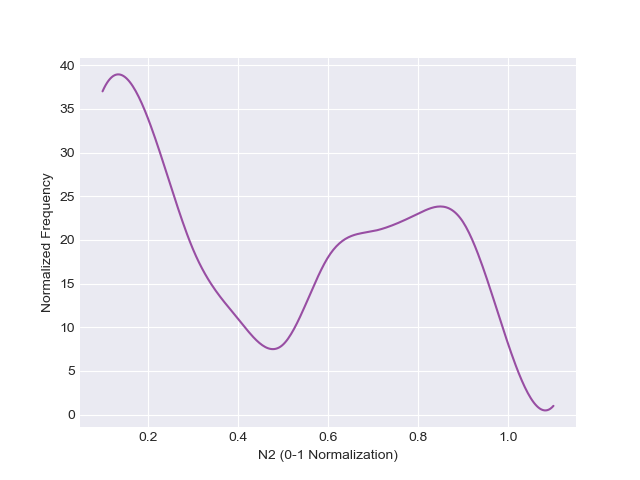


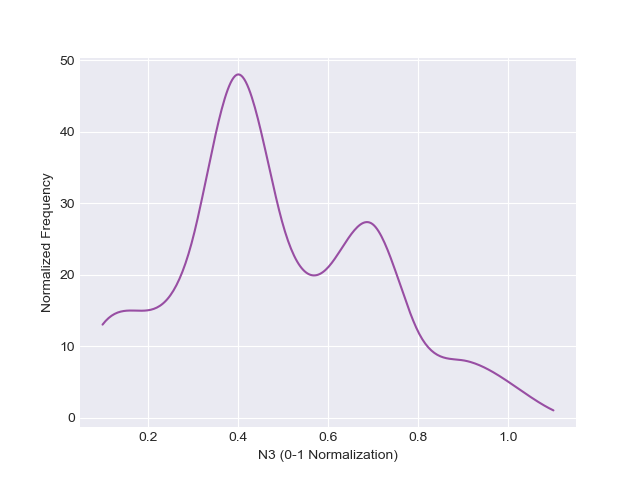

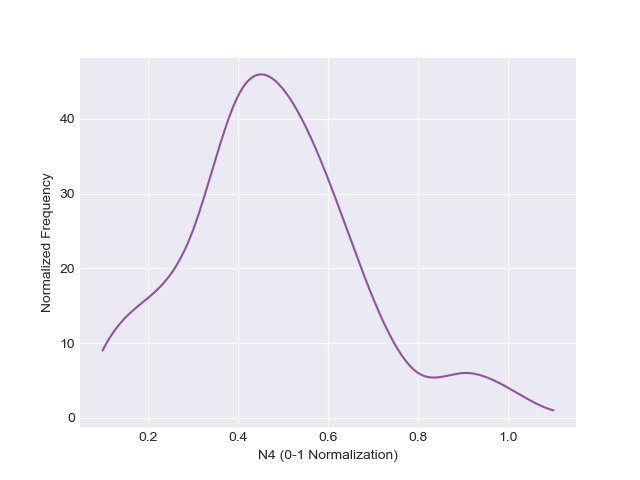


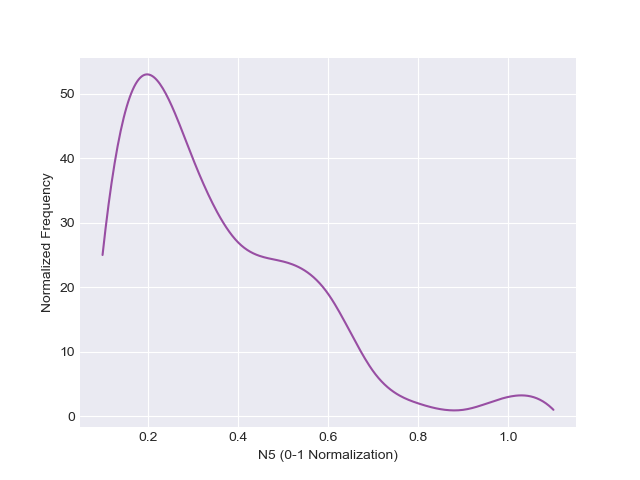

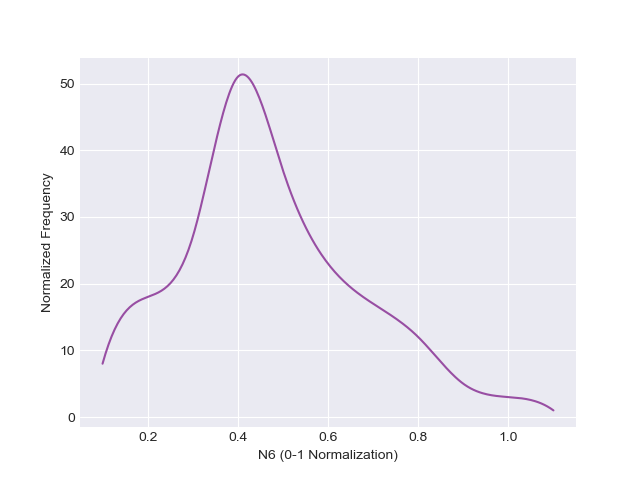


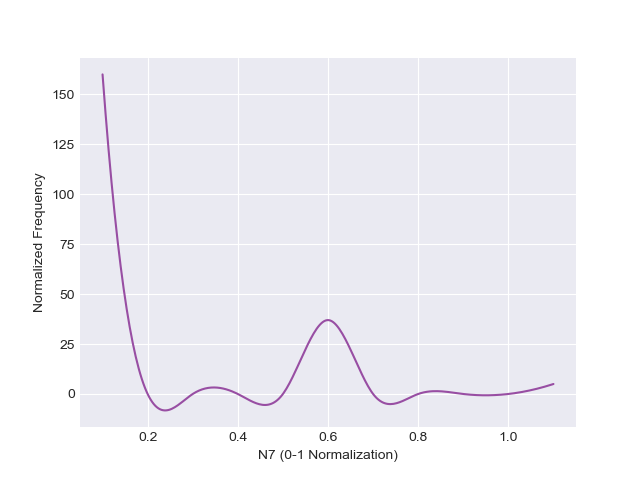

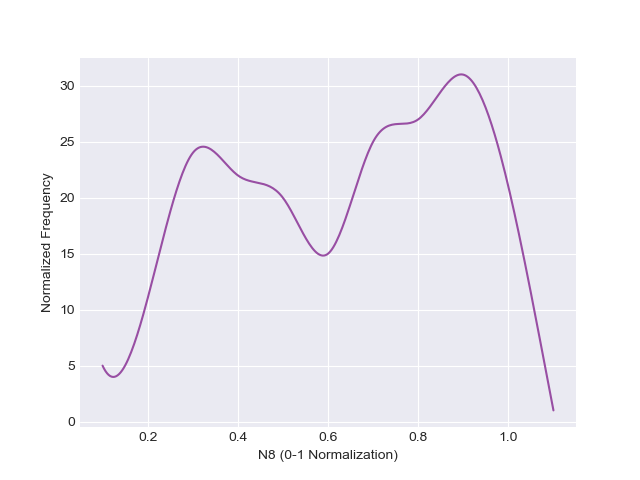


**Appendix A. Normalization**


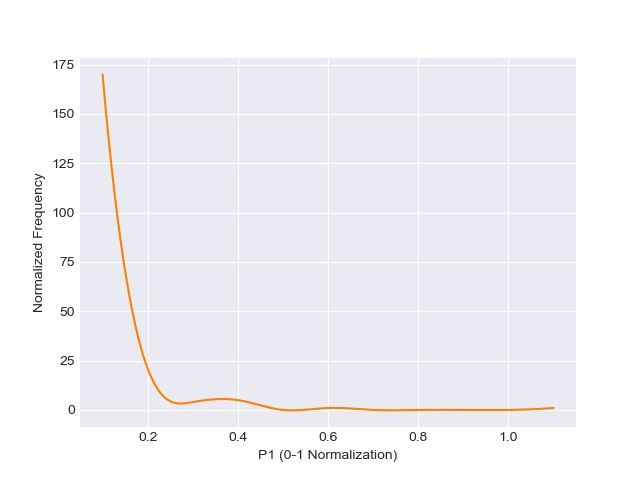

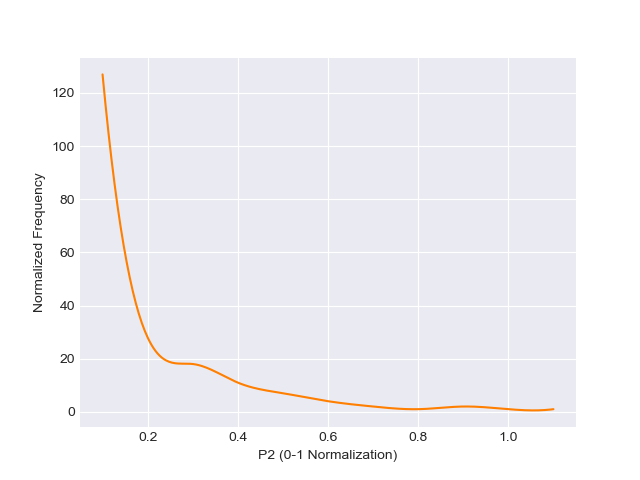


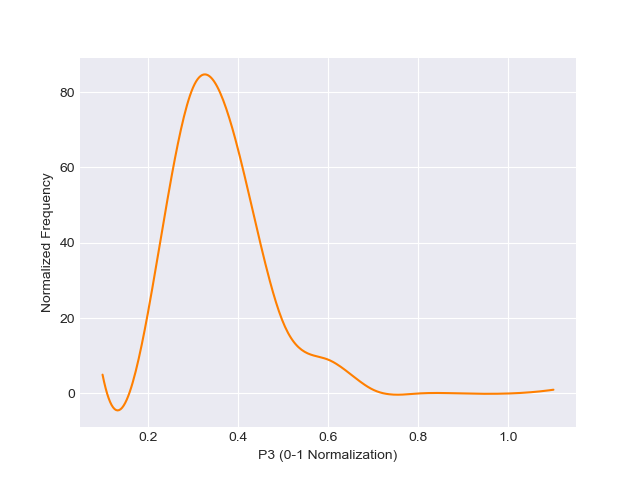

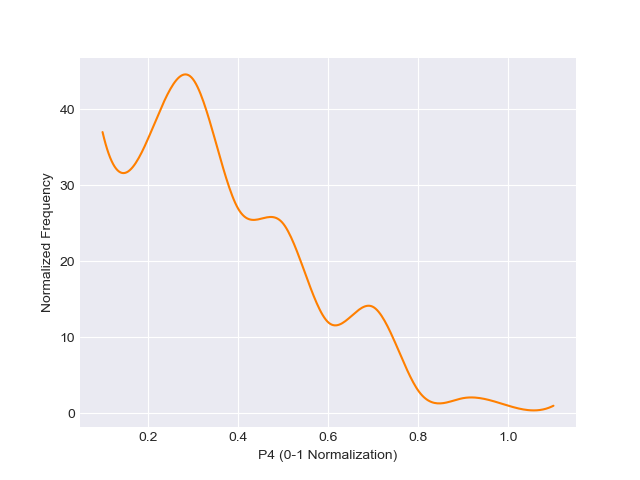


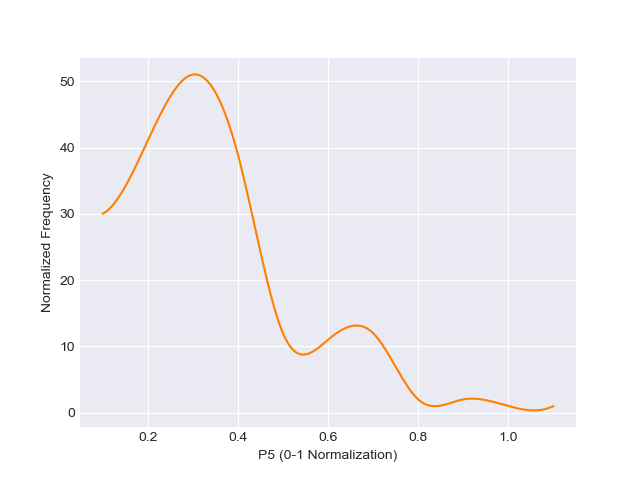

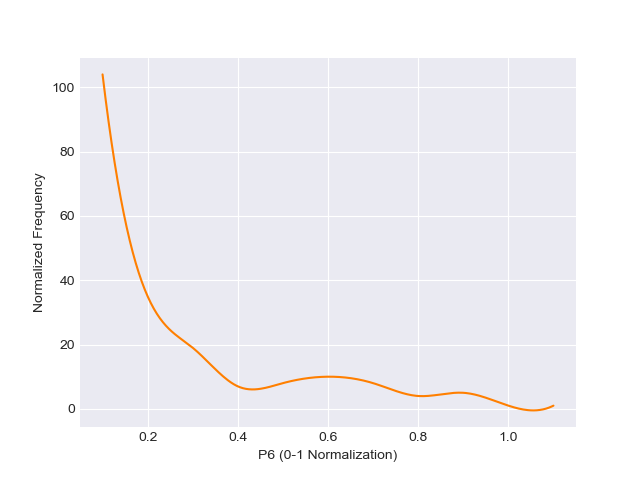


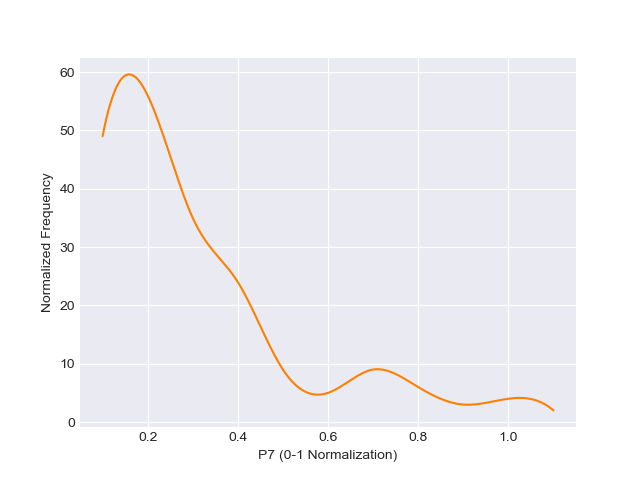

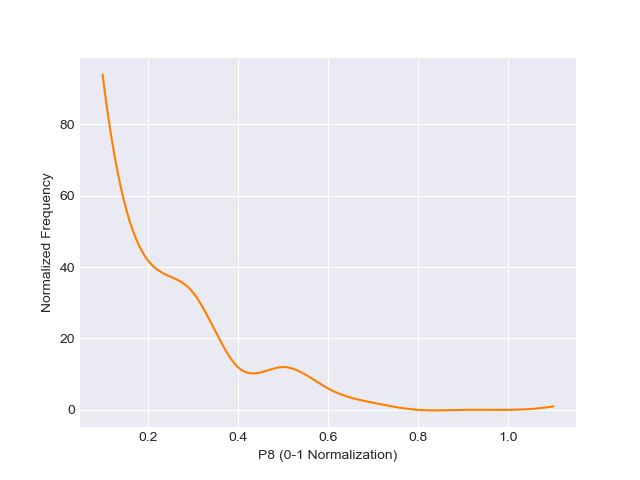


**Appendix A. Normalization**


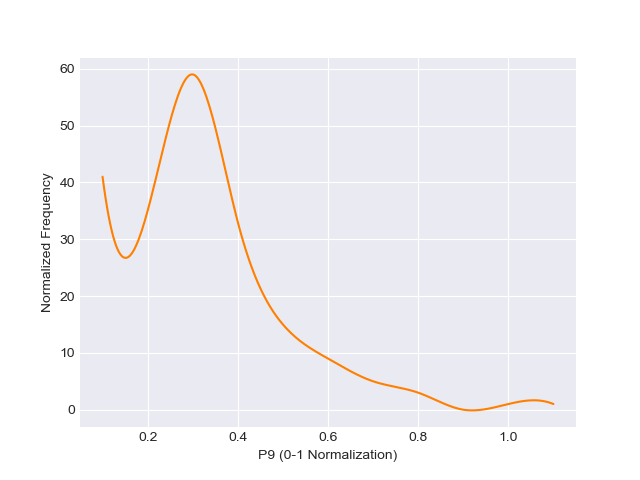

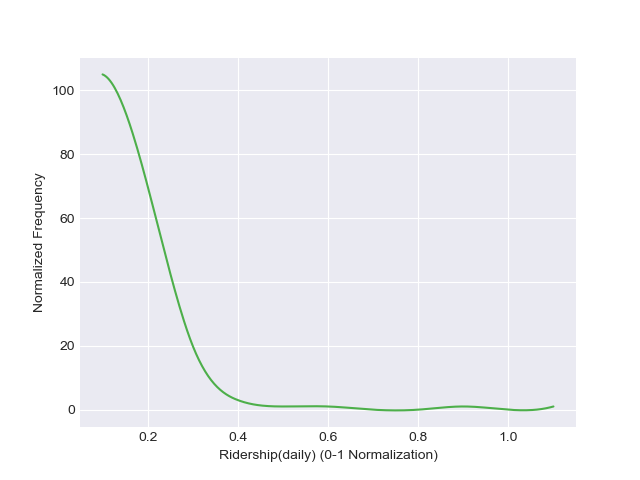


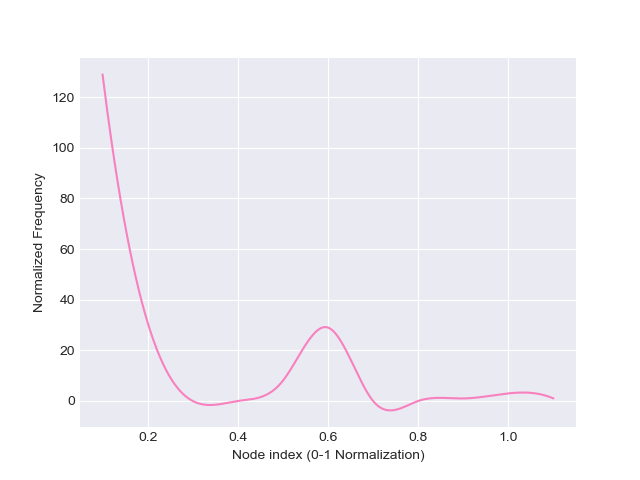

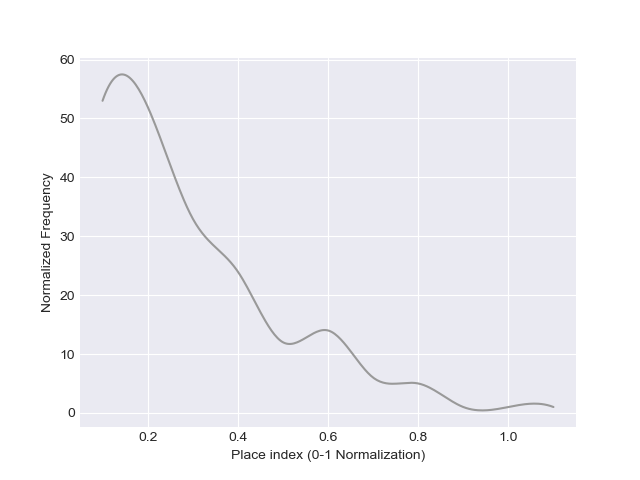


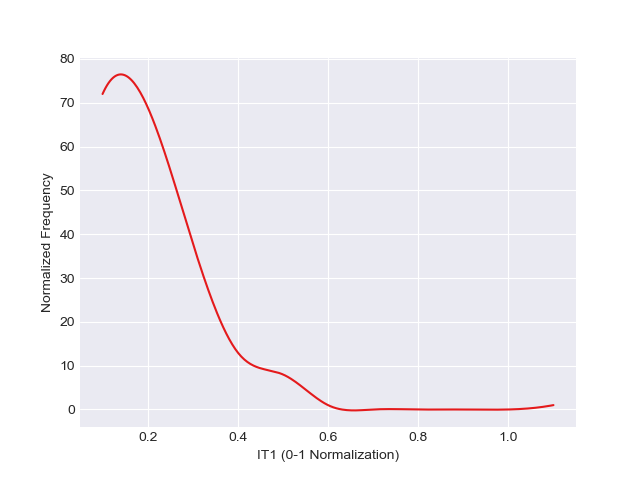

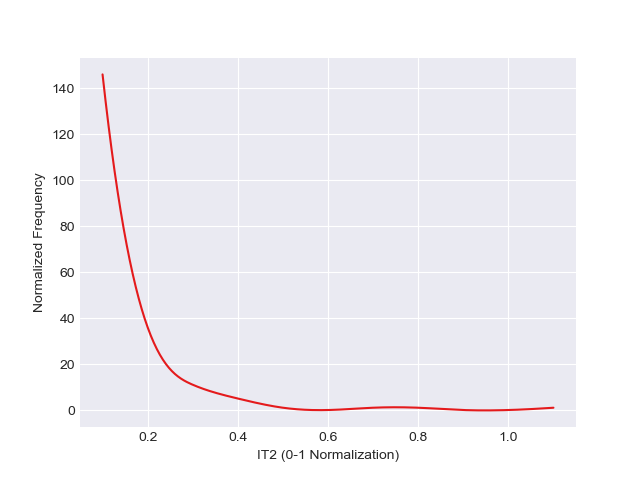


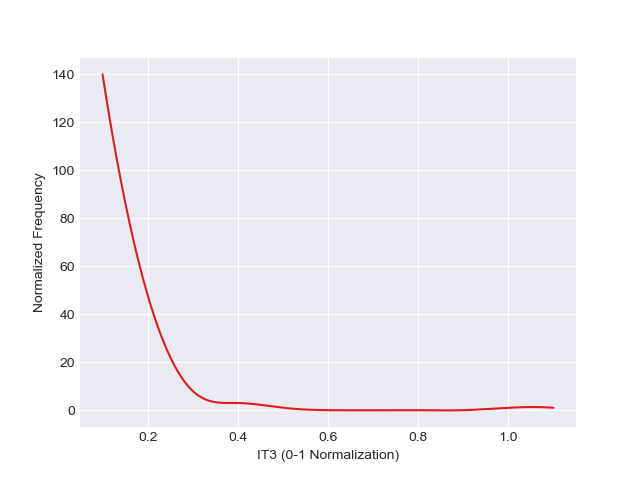

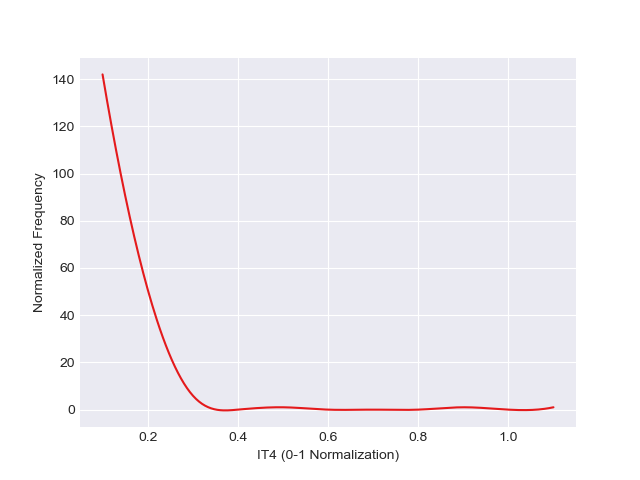


**Appendix A. Normalization**


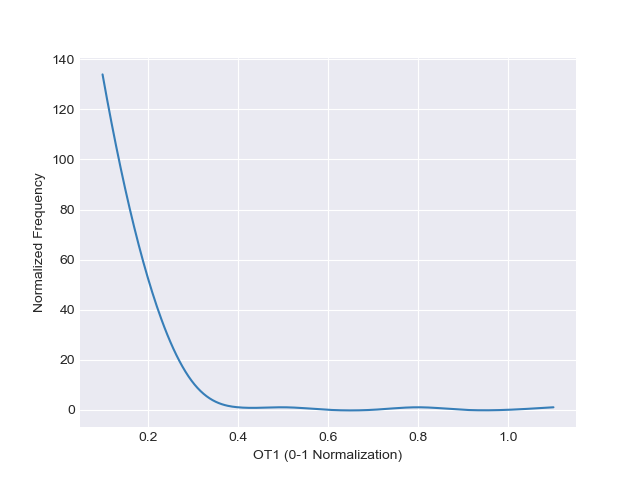

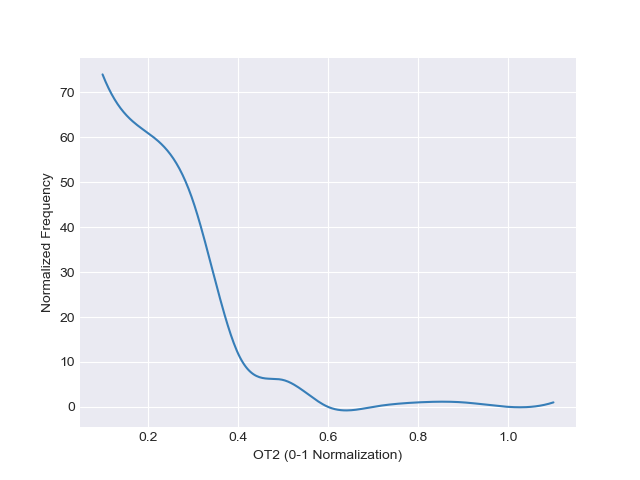


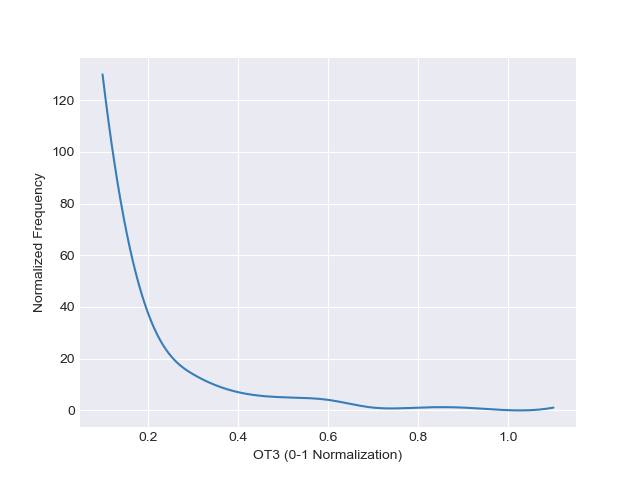

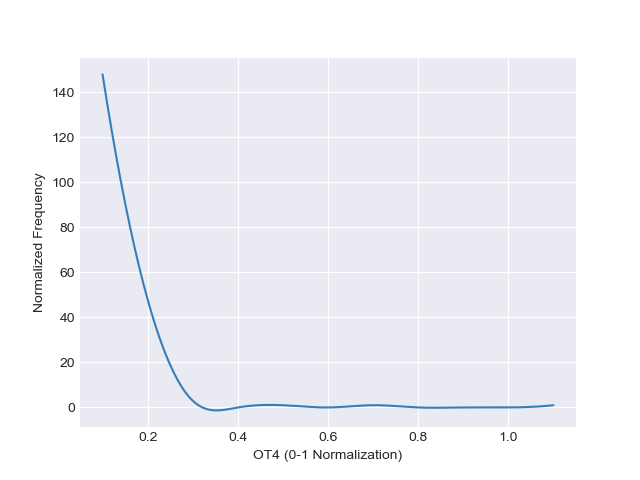


**Appendix B. K-Means method results of NPRT model**


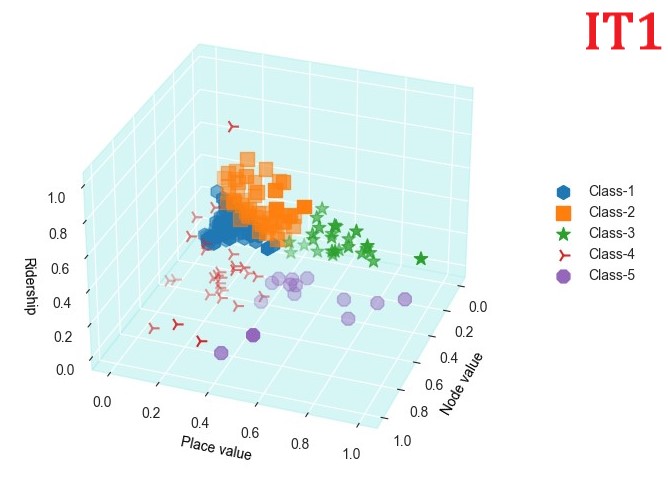

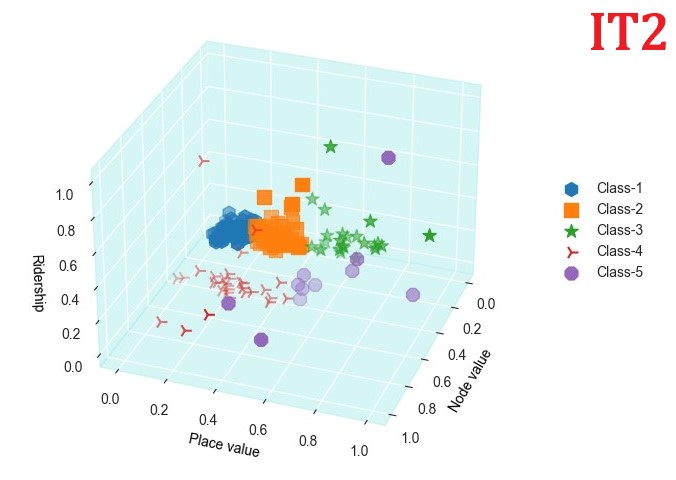


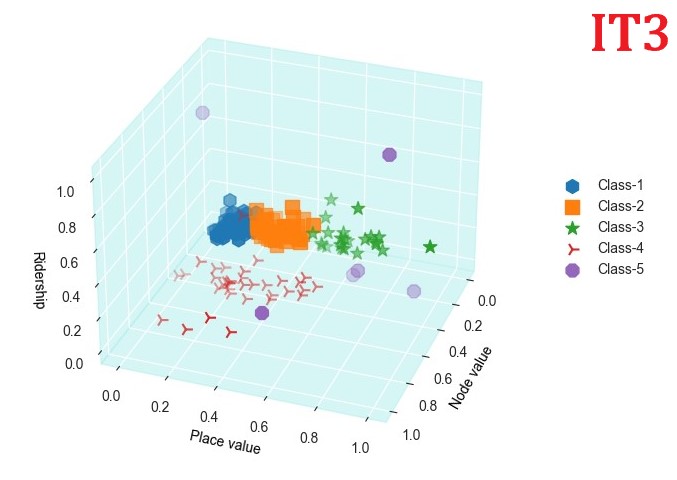

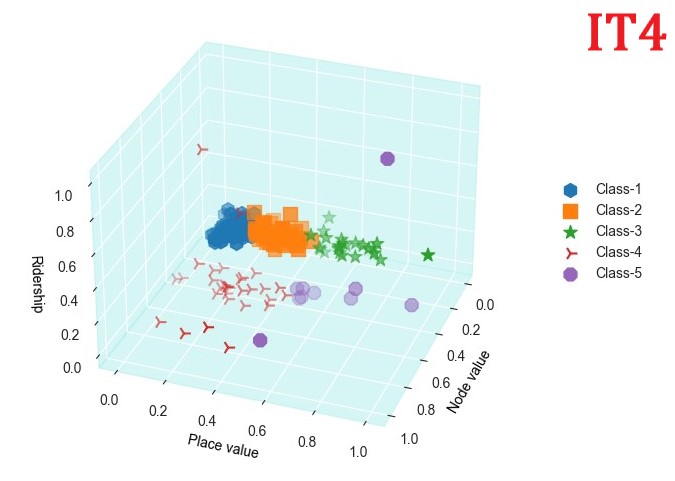


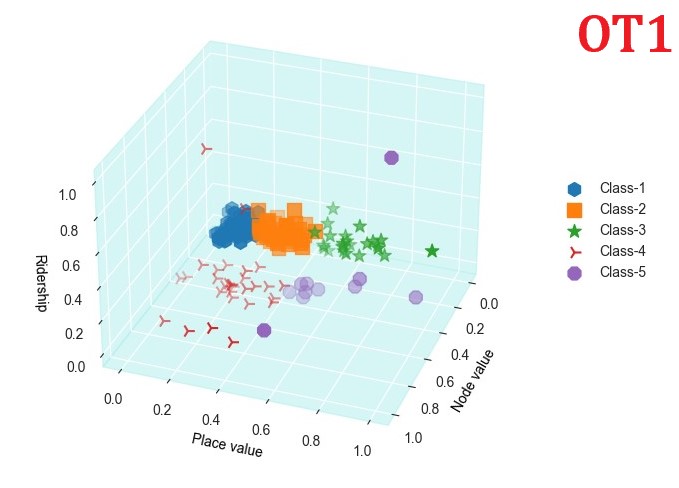

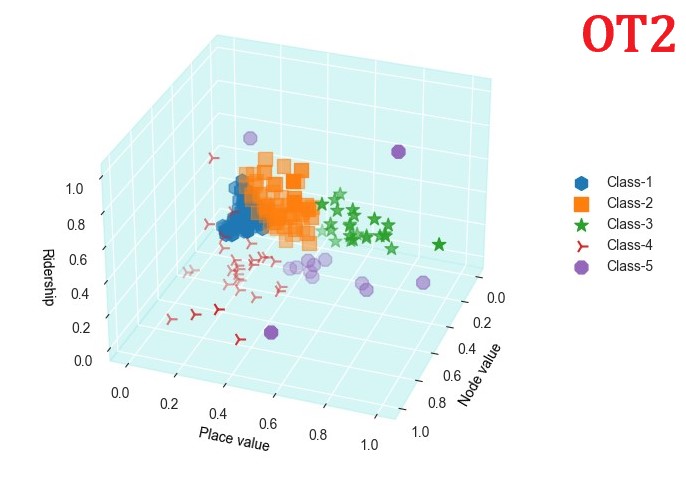


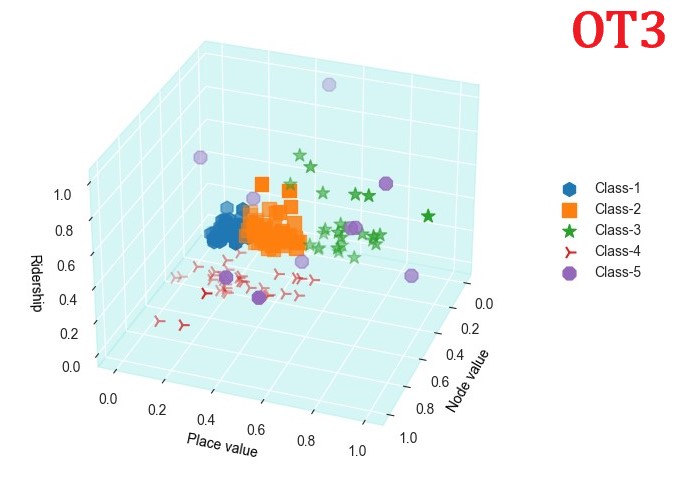

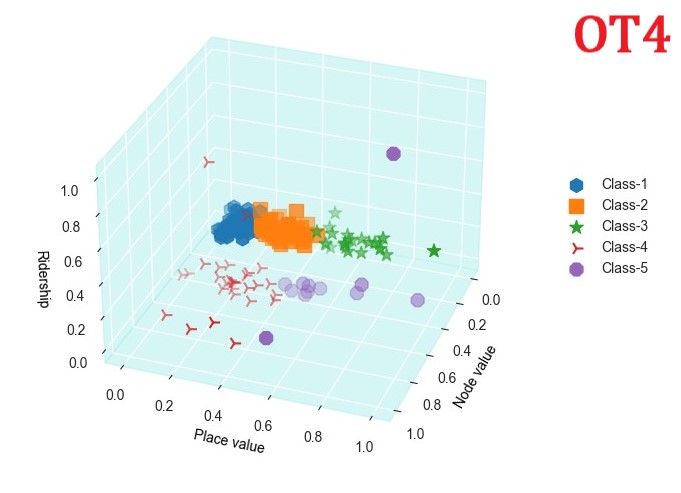


**Appendix C. Cube method results of NPRT model**


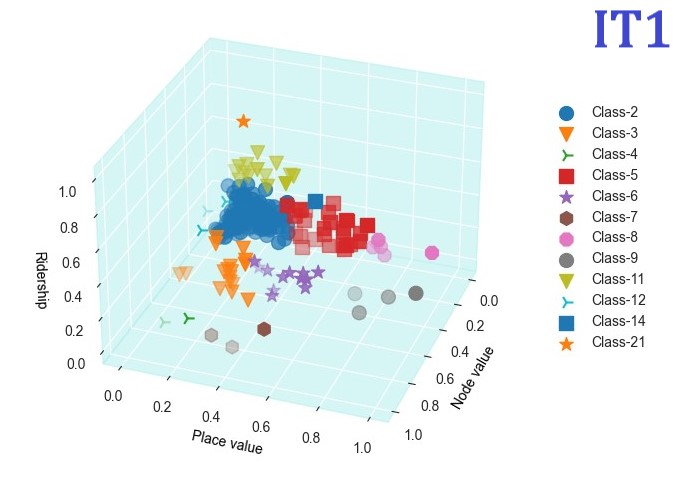

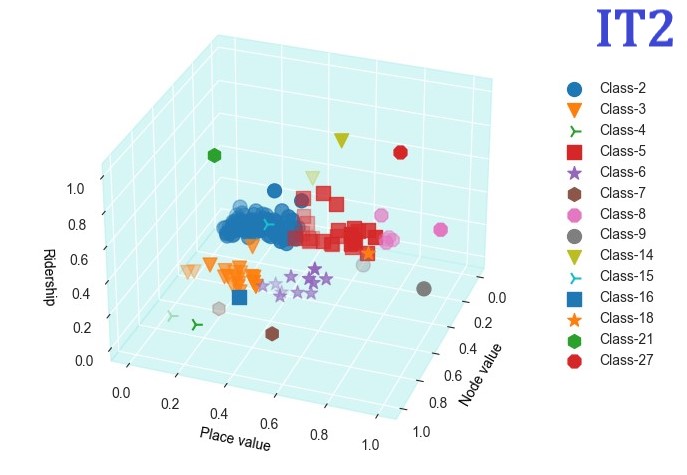


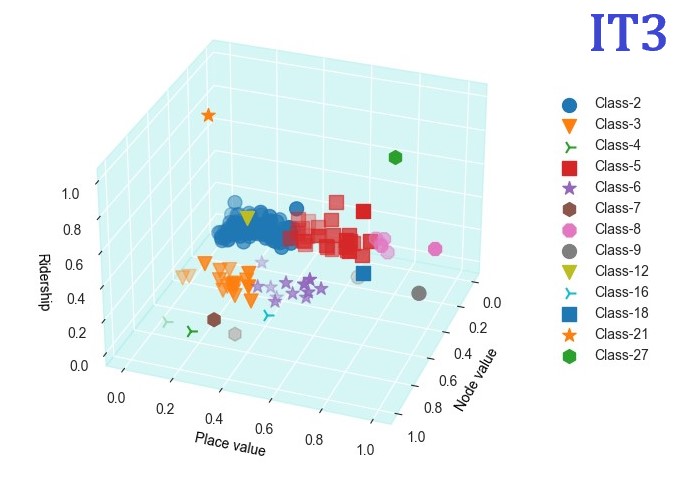

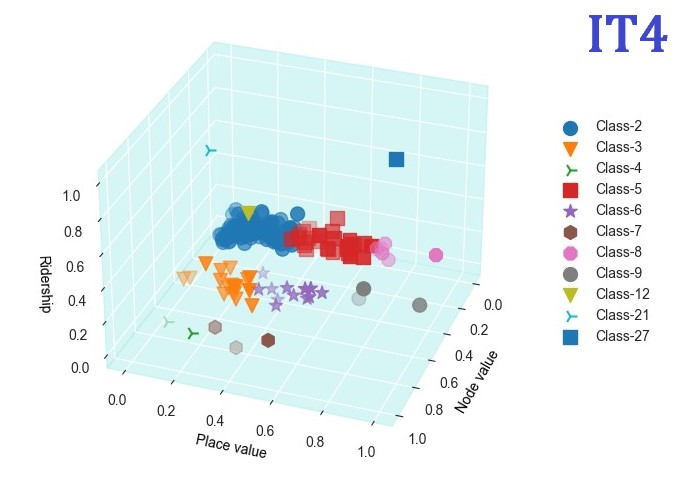


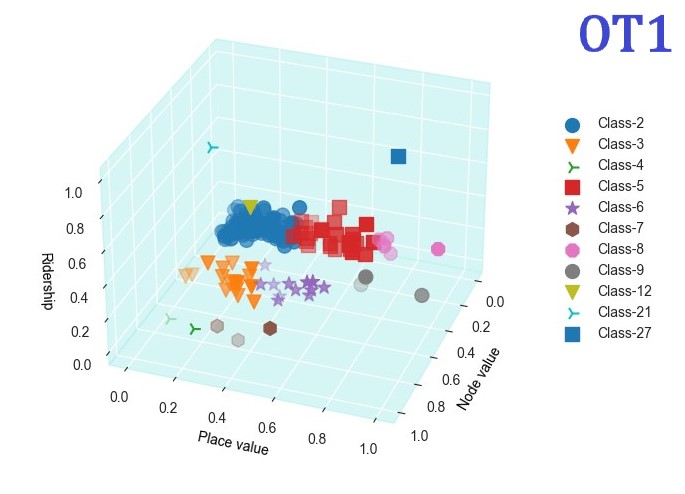

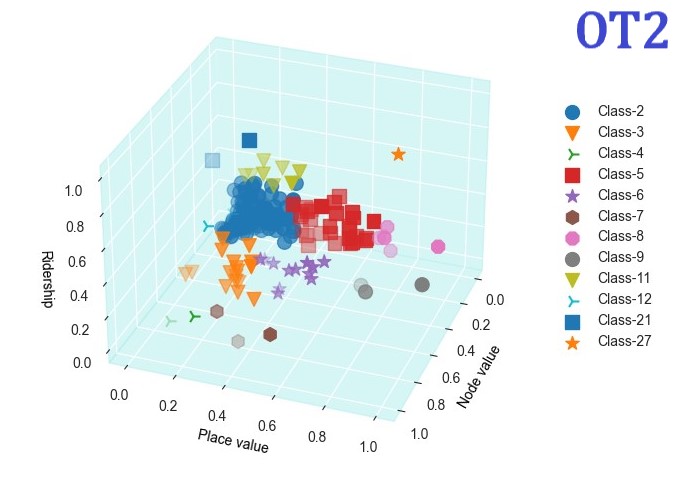


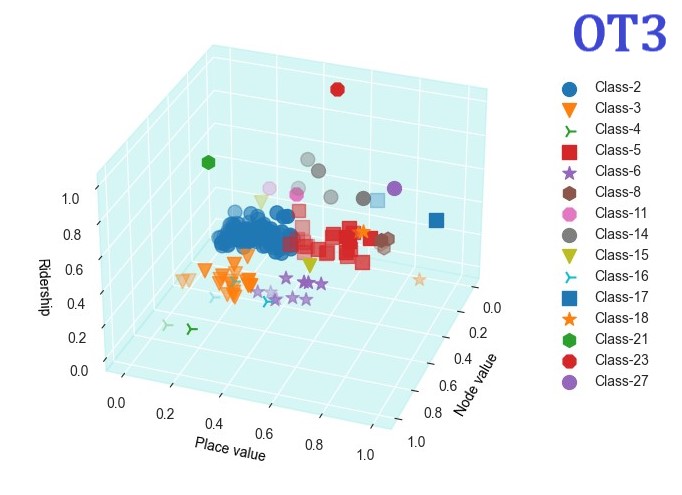

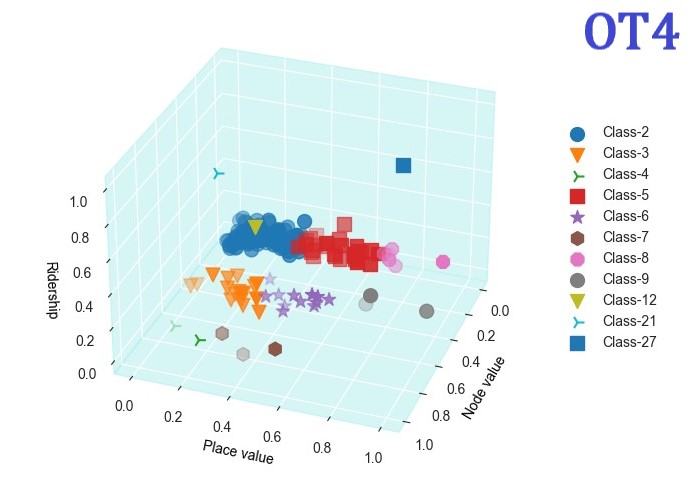


**Appendix D. K-Means method results of Node-Place (NP) model**


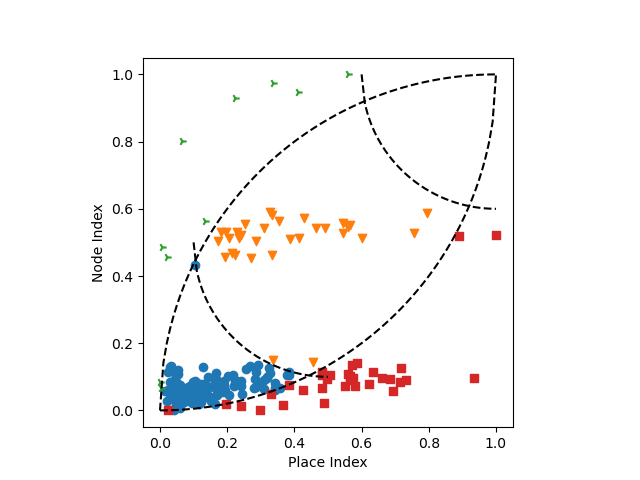


**Appendix E. K-Means method results of Node-Place-Ridership (NPR)**


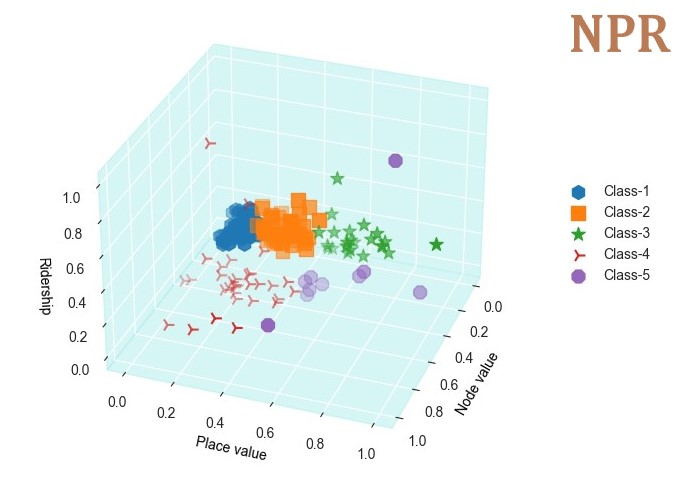


**Appendix F. Stations classification results (NP, NPR, NPRT)**

| **Station** | **Method** | **Node Value (N)** | **Place Value (P)** | **Ridership Value (R)** | **Time Span (T)** | **Class Type** |
| --- | --- | --- | --- | --- | --- | --- |
| Weijianian | NP | 0.0819 | 0.0987 | - | - | 1 |
|  | NPR [K-Means] | 0.0819 | 0.0987 | 0.0768 | - | 1 |
|  | NPRT [K-Means] | 0.0819 | 0.0987 | 0.2506 | IT1 | 2 |
|  | NPRT [Cube] |  |  |  |  | 2 |
|  | NPRT [K-Means] | 0.0819 | 0.0987 | 0.0231 | IT2 | 1 |
|  | NPRT [Cube] |  |  |  |  | 2 |
|  | NPRT [K-Means] | 0.0819 | 0.0987 | 0.0409 | IT3 | 1 |
|  | NPRT [Cube] |  |  |  |  | 2 |
|  | NPRT [K-Means] | 0.0819 | 0.0987 | 0.0509 | IT4 | 1 |
|  | NPRT [Cube] |  |  |  |  | 2 |
|  | NPRT [K-Means] | 0.0819 | 0.0987 | 0.0499 | OT1 | 1 |
|  | NPRT [Cube] |  |  |  |  | 2 |
|  | NPRT [K-Means] | 0.0819 | 0.0987 | 0.1558 | OT2 | 1 |
|  | NPRT [Cube] |  |  |  |  | 2 |
|  | NPRT [K-Means] | 0.0819 | 0.0987 | 0.0233 | OT3 | 1 |
|  | NPRT [Cube] |  |  |  |  | 2 |
|  | NPRT [K-Means] | 0.0819 | 0.0987 | 0.0407 | OT4 | 1 |
|  | NPRT [Cube] |  |  |  |  | 2 |
| Shengxian Lake | NP | 0.0811 | 0.1111 | - | - | 1 |
|  | NPR [K-Means] | 0.0811 | 0.1111 | 0.0507 | - | 1 |
|  | NPRT [K-Means] | 0.0811 | 0.1111 | 0.1132 | IT1 | 1 |
|  | NPRT [Cube] |  |  |  |  | 2 |
|  | NPRT [K-Means] | 0.0811 | 0.1111 | 0.0221 | IT2 | 1 |
|  | NPRT [Cube] |  |  |  |  | 2 |
|  | NPRT [K-Means] | 0.0811 | 0.1111 | 0.0371 | IT3 | 1 |
|  | NPRT [Cube] |  |  |  |  | 2 |
|  | NPRT [K-Means] | 0.0811 | 0.1111 | 0.0323 | IT4 | 1 |
|  | NPRT [Cube] |  |  |  |  | 2 |
|  | NPRT [K-Means] | 0.0811 | 0.1111 | 0.0417 | OT1 | 1 |
|  | NPRT [Cube] |  |  |  |  | 2 |
|  | NPRT [K-Means] | 0.0811 | 0.1111 | 0.0813 | OT2 | 1 |
|  | NPRT [Cube] |  |  |  |  | 2 |
|  | NPRT [K-Means] | 0.0811 | 0.1111 | 0.0343 | OT3 | 1 |
|  | NPRT [Cube] |  |  |  |  | 2 |
|  | NPRT [K-Means] | 0.0811 | 0.1111 | 0.0292 | OT4 | 1 |
|  | NPRT [Cube] |  |  |  |  | 2 |
| Hi-Tech Zone | NP | 0.0681 | 0.3434 | - | - | 1 |
|  | NPR [K-Means] | 0.0681 | 0.3434 | 0.2294 | - | 2 |
|  | NPRT [K-Means] | 0.0681 | 0.3434 | 0.1905 | IT1 | 2 |
|  | NPRT [Cube] |  |  |  |  | 5 |
|  | NPRT [K-Means] | 0.0681 | 0.3434 | 0.2754 | IT2 | 2 |
|  | NPRT [Cube] |  |  |  |  | 5 |
|  | NPRT [K-Means] | 0.0681 | 0.3434 | 0.1779 | IT3 | 2 |
|  | NPRT [Cube] |  |  |  |  | 5 |
|  | NPRT [K-Means] | 0.0681 | 0.3434 | 0.0968 | IT4 | 2 |
|  | NPRT [Cube] |  |  |  |  | 5 |
|  | NPRT [K-Means] | 0.0681 | 0.3434 | 0.1569 | OT1 | 2 |
|  | NPRT [Cube] |  |  |  |  | 5 |
|  | NPRT [K-Means] | 0.0681 | 0.3434 | 0.1859 | OT2 | 2 |
|  | NPRT [Cube] |  |  |  |  | 5 |
|  | NPRT [K-Means] | 0.0681 | 0.3434 | 0.3976 | OT3 | 3 |
|  | NPRT [Cube] |  |  |  |  | 14 |
|  | NPRT [K-Means] | 0.0681 | 0.3434 | 0.0927 | OT4 | 2 |
|  | NPRT [Cube] |  |  |  |  | 5 |
| Jincheng Plaza | NP | 0.0727 | 0.2207 | - | - | 1 |
|  | NPR [K-Means] | 0.0727 | 0.2207 | 0.1996 | - | 2 |
|  | NPRT [K-Means] | 0.0727 | 0.2207 | 0.0303 | IT1 | 1 |
|  | NPRT [Cube] |  |  |  |  | 2 |
|  | NPRT [K-Means] | 0.0727 | 0.2207 | 0.2919 | IT2 | 2 |
|  | NPRT [Cube] |  |  |  |  | 2 |
|  | NPRT [K-Means] | 0.0727 | 0.2207 | 0.1632 | IT3 | 2 |
|  | NPRT [Cube] |  |  |  |  | 2 |
|  | NPRT [K-Means] | 0.0727 | 0.2207 | 0.1294 | IT4 | 2 |
|  | NPRT [Cube] |  |  |  |  | 2 |
|  | NPRT [K-Means] | 0.0727 | 0.2207 | 0.1399 | OT1 | 2 |
|  | NPRT [Cube] |  |  |  |  | 2 |
|  | NPRT [K-Means] | 0.0727 | 0.2207 | 0.0988 | OT2 | 2 |
|  | NPRT [Cube] |  |  |  |  | 2 |
|  | NPRT [K-Means] | 0.0727 | 0.2207 | 0.3689 | OT3 | 2 |
|  | NPRT [Cube] |  |  |  |  | 11 |
|  | NPRT [K-Means] | 0.0727 | 0.2207 | 0.119 | OT4 | 2 |
|  | NPRT [Cube] |  |  |  |  | 2 |

**Appendix F. Stations classification results (NP, NPR, NPRT)**

| **Station** | **Method** | **Node Value (N)** | **Place Value (P)** | **Ridership Value (R)** | **Time Span (T)** | **Class Type** |
| --- | --- | --- | --- | --- | --- | --- |
| Huafu Avenue | NP | 0.0183 | 0.1626 | - | - | 1 |
|  | NPR [K-Means] | 0.0183 | 0.1626 | 0.2001 | - | 2 |
|  | NPRT [K-Means] | 0.0183 | 0.1626 | 0.4931 | IT1 | 2 |
|  | NPRT [Cube] |  |  |  |  | 11 |
|  | NPRT [K-Means] | 0.0183 | 0.1626 | 0.0857 | IT2 | 1 |
|  | NPRT [Cube] |  |  |  |  | 2 |
|  | NPRT [K-Means] | 0.0183 | 0.1626 | 0.119 | IT3 | 1 |
|  | NPRT [Cube] |  |  |  |  | 2 |
|  | NPRT [K-Means] | 0.0183 | 0.1626 | 0.1385 | IT4 | 1 |
|  | NPRT [Cube] |  |  |  |  | 2 |
|  | NPRT [K-Means] | 0.0183 | 0.1626 | 0.1401 | OT1 | 1 |
|  | NPRT [Cube] |  |  |  |  | 2 |
|  | NPRT [K-Means] | 0.0183 | 0.1626 | 0.4375 | OT2 | 2 |
|  | NPRT [Cube] |  |  |  |  | 11 |
|  | NPRT [K-Means] | 0.0183 | 0.1626 | 0.0797 | OT3 | 1 |
|  | NPRT [Cube] |  |  |  |  | 2 |
|  | NPRT [K-Means] | 0.0183 | 0.1626 | 0.1207 | OT4 | 1 |
|  | NPRT [Cube] |  |  |  |  | 2 |
| Honghe | NP | 0.0275 | 0.1126 | - | - | 1 |
|  | NPR [K-Means] | 0.0275 | 0.1126 | 0.179 | - | 1 |
|  | NPRT [K-Means] | 0.0275 | 0.1126 | 0.3624 | IT1 | 2 |
|  | NPRT [Cube] |  |  |  |  | 11 |
|  | NPRT [K-Means] | 0.0275 | 0.1126 | 0.0841 | IT2 | 1 |
|  | NPRT [Cube] |  |  |  |  | 2 |
|  | NPRT [K-Means] | 0.0275 | 0.1126 | 0.1224 | IT3 | 1 |
|  | NPRT [Cube] |  |  |  |  | 2 |
|  | NPRT [K-Means] | 0.0275 | 0.1126 | 0.1339 | IT4 | 1 |
|  | NPRT [Cube] |  |  |  |  | 2 |
|  | NPRT [K-Means] | 0.0275 | 0.1126 | 0.1405 | OT1 | 1 |
|  | NPRT [Cube] |  |  |  |  | 2 |
|  | NPRT [K-Means] | 0.0275 | 0.1126 | 0.3403 | OT2 | 2 |
|  | NPRT [Cube] |  |  |  |  | 11 |
|  | NPRT [K-Means] | 0.0275 | 0.1126 | 0.0968 | OT3 | 1 |
|  | NPRT [Cube] |  |  |  |  | 2 |
|  | NPRT [K-Means] | 0.0275 | 0.1126 | 0.1281 | OT4 | 1 |
|  | NPRT [Cube] |  |  |  |  | 2 |
| East Chengdu Railway Station | NP | 0.4324 | 0.1041 | - | - | 1 |
|  | NPR [K-Means] | 0.4324 | 0.1041 | 0.8496 | - | 4 |
|  | NPRT [K-Means] | 0.4324 | 0.1041 | 0.4104 | IT1 | 4 |
|  | NPRT [Cube] |  |  |  |  | 12 |
|  | NPRT [K-Means] | 0.4324 | 0.1041 | 0.7281 | IT2 | 4 |
|  | NPRT [Cube] |  |  |  |  | 21 |
|  | NPRT [K-Means] | 0.4324 | 0.1041 | 0.9922 | IT3 | 5 |
|  | NPRT [Cube] |  |  |  |  | 21 |
|  | NPRT [K-Means] | 0.4324 | 0.1041 | 0.802 | IT4 | 4 |
|  | NPRT [Cube] |  |  |  |  | 21 |
|  | NPRT [K-Means] | 0.4324 | 0.1041 | 0.7995 | OT1 | 4 |
|  | NPRT [Cube] |  |  |  |  | 21 |
|  | NPRT [K-Means] | 0.4324 | 0.1041 | 0.7102 | OT2 | 4 |
|  | NPRT [Cube] |  |  |  |  | 21 |
|  | NPRT [K-Means] | 0.4324 | 0.1041 | 0.749 | OT3 | 5 |
|  | NPRT [Cube] |  |  |  |  | 21 |
|  | NPRT [K-Means] | 0.4324 | 0.1041 | 0.6982 | OT4 | 4 |
|  | NPRT [Cube] |  |  |  |  | 21 |
| Fenghuang Street | NP | 0.0564 | 0.0961 | - | - | 1 |
|  | NPR [K-Means] | 0.0564 | 0.0961 | 0.1634 | - | 1 |
|  | NPRT [K-Means] | 0.0564 | 0.0961 | 0.4169 | IT1 | 2 |
|  | NPRT [Cube] |  |  |  |  | 11 |
|  | NPRT [K-Means] | 0.0564 | 0.0961 | 0.0546 | IT2 | 1 |
|  | NPRT [Cube] |  |  |  |  | 2 |
|  | NPRT [K-Means] | 0.0564 | 0.0961 | 0.0959 | IT3 | 1 |
|  | NPRT [Cube] |  |  |  |  | 2 |
|  | NPRT [K-Means] | 0.0564 | 0.0961 | 0.1211 | IT4 | 1 |
|  | NPRT [Cube] |  |  |  |  | 2 |
|  | NPRT [K-Means] | 0.0564 | 0.0961 | 0.1248 | OT1 | 1 |
|  | NPRT [Cube] |  |  |  |  | 2 |
|  | NPRT [K-Means] | 0.0564 | 0.0961 | 0.3545 | OT2 | 2 |
|  | NPRT [Cube] |  |  |  |  | 11 |
|  | NPRT [K-Means] | 0.0564 | 0.0961 | 0.0513 | OT3 | 1 |
|  | NPRT [Cube] |  |  |  |  | 2 |
|  | NPRT [K-Means] | 0.0564 | 0.0961 | 0.1088 | OT4 | 1 |
|  | NPRT [Cube] |  |  |  |  | 2 |

**Appendix F. Stations classification results (NP, NPR, NPRT)**

| **Station** | **Method** | **Node Value (N)** | **Place Value (P)** | **Ridership Value (R)** | **Time Span (T)** | **Class Type** |
| --- | --- | --- | --- | --- | --- | --- |
| Qingyang Taoist Temple | NP | 0.1056 | 0.3852 | - | - | 1 |
|  | NPR [K-Means] | 0.1056 | 0.3852 | 0.0515 | - | 2 |
|  | NPRT [K-Means] | 0.1056 | 0.3852 | 0.0279 | IT1 | 3 |
|  | NPRT [Cube] |  |  |  |  | 5 |
|  | NPRT [K-Means] | 0.1056 | 0.3852 | 0.0481 | IT2 | 2 |
|  | NPRT [Cube] |  |  |  |  | 5 |
|  | NPRT [K-Means] | 0.1056 | 0.3852 | 0.055 | IT3 | 2 |
|  | NPRT [Cube] |  |  |  |  | 5 |
|  | NPRT [K-Means] | 0.1056 | 0.3852 | 0.0369 | IT4 | 2 |
|  | NPRT [Cube] |  |  |  |  | 5 |
|  | NPRT [K-Means] | 0.1056 | 0.3852 | 0.043 | OT1 | 2 |
|  | NPRT [Cube] |  |  |  |  | 5 |
|  | NPRT [K-Means] | 0.1056 | 0.3852 | 0.0316 | OT2 | 2 |
|  | NPRT [Cube] |  |  |  |  | 5 |
|  | NPRT [K-Means] | 0.1056 | 0.3852 | 0.0763 | OT3 | 2 |
|  | NPRT [Cube] |  |  |  |  | 5 |
|  | NPRT [K-Means] | 0.1056 | 0.3852 | 0.0346 | OT4 | 2 |
|  | NPRT [Cube] |  |  |  |  | 5 |
| Provincial Orthopaedics Hospital | NP | 0.1049 | 0.3781 | - | - | 1 |
|  | NPR [K-Means] | 0.1049 | 0.3781 | 0.0848 | - | 2 |
|  | NPRT [K-Means] | 0.1049 | 0.3781 | 0.0917 | IT1 | 3 |
|  | NPRT [Cube] |  |  |  |  | 5 |
|  | NPRT [K-Means] | 0.1049 | 0.3781 | 0.0633 | IT2 | 2 |
|  | NPRT [Cube] |  |  |  |  | 5 |
|  | NPRT [K-Means] | 0.1049 | 0.3781 | 0.0775 | IT3 | 2 |
|  | NPRT [Cube] |  |  |  |  | 5 |
|  | NPRT [K-Means] | 0.1049 | 0.3781 | 0.0595 | IT4 | 2 |
|  | NPRT [Cube] |  |  |  |  | 5 |
|  | NPRT [K-Means] | 0.1049 | 0.3781 | 0.0691 | OT1 | 2 |
|  | NPRT [Cube] |  |  |  |  | 5 |
|  | NPRT [K-Means] | 0.1049 | 0.3781 | 0.0951 | OT2 | 2 |
|  | NPRT [Cube] |  |  |  |  | 5 |
|  | NPRT [K-Means] | 0.1049 | 0.3781 | 0.1014 | OT3 | 2 |
|  | NPRT [Cube] |  |  |  |  | 5 |
|  | NPRT [K-Means] | 0.1049 | 0.3781 | 0.0543 | OT4 | 2 |
|  | NPRT [Cube] |  |  |  |  | 5 |
| Chengdu First People's Hospital | NP | 0.0612 | 0.3413 | - | - | 1 |
|  | NPR [K-Means] | 0.0612 | 0.3413 | 0.1268 | - | 2 |
|  | NPRT [K-Means] | 0.0612 | 0.3413 | 0.0674 | IT1 | 2 |
|  | NPRT [Cube] |  |  |  |  | 5 |
|  | NPRT [K-Means] | 0.0612 | 0.3413 | 0.1577 | IT2 | 2 |
|  | NPRT [Cube] |  |  |  |  | 5 |
|  | NPRT [K-Means] | 0.0612 | 0.3413 | 0.1011 | IT3 | 2 |
|  | NPRT [Cube] |  |  |  |  | 5 |
|  | NPRT [K-Means] | 0.0612 | 0.3413 | 0.0721 | IT4 | 2 |
|  | NPRT [Cube] |  |  |  |  | 5 |
|  | NPRT [K-Means] | 0.0612 | 0.3413 | 0.0768 | OT1 | 2 |
|  | NPRT [Cube] |  |  |  |  | 5 |
|  | NPRT [K-Means] | 0.0612 | 0.3413 | 0.0804 | OT2 | 2 |
|  | NPRT [Cube] |  |  |  |  | 5 |
|  | NPRT [K-Means] | 0.0612 | 0.3413 | 0.2549 | OT3 | 2 |
|  | NPRT [Cube] |  |  |  |  | 5 |
|  | NPRT [K-Means] | 0.0612 | 0.3413 | 0.0663 | OT4 | 2 |
|  | NPRT [Cube] |  |  |  |  | 5 |
| Wugensong | NP | 0.0192 | 0.1155 | - | - | 1 |
|  | NPR [K-Means] | 0.0192 | 0.1155 | 0.1181 | - | 1 |
|  | NPRT [K-Means] | 0.0192 | 0.1155 | 0.4099 | IT1 | 2 |
|  | NPRT [Cube] |  |  |  |  | 11 |
|  | NPRT [K-Means] | 0.0192 | 0.1155 | 0.0345 | IT2 | 1 |
|  | NPRT [Cube] |  |  |  |  | 2 |
|  | NPRT [K-Means] | 0.0192 | 0.1155 | 0.0532 | IT3 | 1 |
|  | NPRT [Cube] |  |  |  |  | 2 |
|  | NPRT [K-Means] | 0.0192 | 0.1155 | 0.0738 | IT4 | 1 |
|  | NPRT [Cube] |  |  |  |  | 2 |
|  | NPRT [K-Means] | 0.0192 | 0.1155 | 0.0665 | OT1 | 1 |
|  | NPRT [Cube] |  |  |  |  | 2 |
|  | NPRT [K-Means] | 0.0192 | 0.1155 | 0.2767 | OT2 | 2 |
|  | NPRT [Cube] |  |  |  |  | 2 |
|  | NPRT [K-Means] | 0.0192 | 0.1155 | 0.0277 | OT3 | 1 |
|  | NPRT [Cube] |  |  |  |  | 2 |
|  | NPRT [K-Means] | 0.0192 | 0.1155 | 0.0536 | OT4 | 1 |
|  | NPRT [Cube] |  |  |  |  | 2 |

**Appendix F. Stations classification results (NP, NPR, NPRT)**

| **Station** | **Method** | **Node Value (N)** | **Place Value (P)** | **Ridership Value (R)** | **Time Span (T)** | **Class Type** |
| --- | --- | --- | --- | --- | --- | --- |
| North Railway Station | NP | 0.5441 | 0.3103 | - | - | 2 |
|  | NPR [K-Means] | 0.5441 | 0.3103 | 0.2063 | - | 4 |
|  | NPRT [K-Means] | 0.5441 | 0.3103 | 0.2507 | IT1 | 4 |
|  | NPRT [Cube] |  |  |  |  | 3 |
|  | NPRT [K-Means] | 0.5441 | 0.3103 | 0.1292 | IT2 | 4 |
|  | NPRT [Cube] |  |  |  |  | 3 |
|  | NPRT [K-Means] | 0.5441 | 0.3103 | 0.1828 | IT3 | 4 |
|  | NPRT [Cube] |  |  |  |  | 3 |
|  | NPRT [K-Means] | 0.5441 | 0.3103 | 0.1682 | IT4 | 4 |
|  | NPRT [Cube] |  |  |  |  | 3 |
|  | NPRT [K-Means] | 0.5441 | 0.3103 | 0.204 | OT1 | 4 |
|  | NPRT [Cube] |  |  |  |  | 3 |
|  | NPRT [K-Means] | 0.5441 | 0.3103 | 0.2335 | OT2 | 4 |
|  | NPRT [Cube] |  |  |  |  | 3 |
|  | NPRT [K-Means] | 0.5441 | 0.3103 | 0.1712 | OT3 | 4 |
|  | NPRT [Cube] |  |  |  |  | 3 |
|  | NPRT [K-Means] | 0.5441 | 0.3103 | 0.1671 | OT4 | 4 |
|  | NPRT [Cube] |  |  |  |  | 3 |
| Renmin North Road | NP | 0.5415 | 0.4643 | - | - | 2 |
|  | NPR [K-Means] | 0.5415 | 0.4643 | 0.1957 | - | 4 |
|  | NPRT [K-Means] | 0.5415 | 0.4643 | 0.19 | IT1 | 5 |
|  | NPRT [Cube] |  |  |  |  | 6 |
|  | NPRT [K-Means] | 0.5415 | 0.4643 | 0.1732 | IT2 | 4 |
|  | NPRT [Cube] |  |  |  |  | 6 |
|  | NPRT [K-Means] | 0.5415 | 0.4643 | 0.1661 | IT3 | 4 |
|  | NPRT [Cube] |  |  |  |  | 6 |
|  | NPRT [K-Means] | 0.5415 | 0.4643 | 0.1483 | IT4 | 4 |
|  | NPRT [Cube] |  |  |  |  | 6 |
|  | NPRT [K-Means] | 0.5415 | 0.4643 | 0.155 | OT1 | 4 |
|  | NPRT [Cube] |  |  |  |  | 6 |
|  | NPRT [K-Means] | 0.5415 | 0.4643 | 0.2221 | OT2 | 5 |
|  | NPRT [Cube] |  |  |  |  | 6 |
|  | NPRT [K-Means] | 0.5415 | 0.4643 | 0.225 | OT3 | 4 |
|  | NPRT [Cube] |  |  |  |  | 6 |
|  | NPRT [K-Means] | 0.5415 | 0.4643 | 0.1387 | OT4 | 5 |
|  | NPRT [Cube] |  |  |  |  | 6 |
| Luomashi | NP | 0.527 | 0.7553 | - | - | 2 |
|  | NPR [K-Means] | 0.527 | 0.7553 | 0.2994 | - | 5 |
|  | NPRT [K-Means] | 0.527 | 0.7553 | 0.1584 | IT1 | 5 |
|  | NPRT [Cube] |  |  |  |  | 9 |
|  | NPRT [K-Means] | 0.527 | 0.7553 | 0.3132 | IT2 | 5 |
|  | NPRT [Cube] |  |  |  |  | 9 |
|  | NPRT [K-Means] | 0.527 | 0.7553 | 0.2707 | IT3 | 5 |
|  | NPRT [Cube] |  |  |  |  | 9 |
|  | NPRT [K-Means] | 0.527 | 0.7553 | 0.1533 | IT4 | 5 |
|  | NPRT [Cube] |  |  |  |  | 9 |
|  | NPRT [K-Means] | 0.527 | 0.7553 | 0.2191 | OT1 | 5 |
|  | NPRT [Cube] |  |  |  |  | 9 |
|  | NPRT [K-Means] | 0.527 | 0.7553 | 0.2026 | OT2 | 5 |
|  | NPRT [Cube] |  |  |  |  | 9 |
|  | NPRT [K-Means] | 0.527 | 0.7553 | 0.566 | OT3 | 5 |
|  | NPRT [Cube] |  |  |  |  | 18 |
|  | NPRT [K-Means] | 0.527 | 0.7553 | 0.1559 | OT4 | 5 |
|  | NPRT [Cube] |  |  |  |  | 9 |
| Tianfu Square | NP | 0.5869 | 0.7938 | - | - | 2 |
|  | NPR [K-Means] | 0.5869 | 0.7938 | 0.3821 | - | 5 |
|  | NPRT [K-Means] | 0.5869 | 0.7938 | 0.1006 | IT1 | 5 |
|  | NPRT [Cube] |  |  |  |  | 9 |
|  | NPRT [K-Means] | 0.5869 | 0.7938 | 0.4388 | IT2 | 5 |
|  | NPRT [Cube] |  |  |  |  | 18 |
|  | NPRT [K-Means] | 0.5869 | 0.7938 | 0.3528 | IT3 | 5 |
|  | NPRT [Cube] |  |  |  |  | 18 |
|  | NPRT [K-Means] | 0.5869 | 0.7938 | 0.269 | IT4 | 5 |
|  | NPRT [Cube] |  |  |  |  | 9 |
|  | NPRT [K-Means] | 0.5869 | 0.7938 | 0.3215 | OT1 | 5 |
|  | NPRT [Cube] |  |  |  |  | 9 |
|  | NPRT [K-Means] | 0.5869 | 0.7938 | 0.2206 | OT2 | 5 |
|  | NPRT [Cube] |  |  |  |  | 9 |
|  | NPRT [K-Means] | 0.5869 | 0.7938 | 0.6221 | OT3 | 5 |
|  | NPRT [Cube] |  |  |  |  | 18 |
|  | NPRT [K-Means] | 0.5869 | 0.7938 | 0.2636 | OT4 | 5 |
|  | NPRT [Cube] |  |  |  |  | 9 |

**Appendix F. Stations classification results (NP, NPR, NPRT)**

| **Station** | **Method** | **Node Value (N)** | **Place Value (P)** | **Ridership Value (R)** | **Time Span (T)** | **Class Type** |
| --- | --- | --- | --- | --- | --- | --- |
| Sichuan Gymnasium | NP | 0.5505 | 0.5661 | - | - | 2 |
|  | NPR [K-Means] | 0.5505 | 0.5661 | 0.2595 | - | 5 |
|  | NPRT [K-Means] | 0.5505 | 0.5661 | 0.2108 | IT1 | 5 |
|  | NPRT [Cube] |  |  |  |  | 6 |
|  | NPRT [K-Means] | 0.5505 | 0.5661 | 0.254 | IT2 | 5 |
|  | NPRT [Cube] |  |  |  |  | 6 |
|  | NPRT [K-Means] | 0.5505 | 0.5661 | 0.2207 | IT3 | 4 |
|  | NPRT [Cube] |  |  |  |  | 6 |
|  | NPRT [K-Means] | 0.5505 | 0.5661 | 0.1801 | IT4 | 5 |
|  | NPRT [Cube] |  |  |  |  | 6 |
|  | NPRT [K-Means] | 0.5505 | 0.5661 | 0.202 | OT1 | 5 |
|  | NPRT [Cube] |  |  |  |  | 6 |
|  | NPRT [K-Means] | 0.5505 | 0.5661 | 0.2784 | OT2 | 5 |
|  | NPRT [Cube] |  |  |  |  | 6 |
|  | NPRT [K-Means] | 0.5505 | 0.5661 | 0.3345 | OT3 | 5 |
|  | NPRT [Cube] |  |  |  |  | 15 |
|  | NPRT [K-Means] | 0.5505 | 0.5661 | 0.1655 | OT4 | 5 |
|  | NPRT [Cube] |  |  |  |  | 6 |
| Nijiaqiao | NP | 0.5145 | 0.6 | - | - | 2 |
|  | NPR [K-Means] | 0.5145 | 0.6 | 0.199 | - | 5 |
|  | NPRT [K-Means] | 0.5145 | 0.6 | 0.2355 | IT1 | 5 |
|  | NPRT [Cube] |  |  |  |  | 6 |
|  | NPRT [K-Means] | 0.5145 | 0.6 | 0.176 | IT2 | 5 |
|  | NPRT [Cube] |  |  |  |  | 6 |
|  | NPRT [K-Means] | 0.5145 | 0.6 | 0.1475 | IT3 | 4 |
|  | NPRT [Cube] |  |  |  |  | 6 |
|  | NPRT [K-Means] | 0.5145 | 0.6 | 0.1346 | IT4 | 5 |
|  | NPRT [Cube] |  |  |  |  | 6 |
|  | NPRT [K-Means] | 0.5145 | 0.6 | 0.1491 | OT1 | 5 |
|  | NPRT [Cube] |  |  |  |  | 6 |
|  | NPRT [K-Means] | 0.5145 | 0.6 | 0.2922 | OT2 | 5 |
|  | NPRT [Cube] |  |  |  |  | 6 |
|  | NPRT [K-Means] | 0.5145 | 0.6 | 0.2061 | OT3 | 4 |
|  | NPRT [Cube] |  |  |  |  | 6 |
|  | NPRT [K-Means] | 0.5145 | 0.6 | 0.1301 | OT4 | 5 |
|  | NPRT [Cube] |  |  |  |  | 6 |
| Century City | NP | 0.463 | 0.3343 | - | - | 2 |
|  | NPR [K-Means] | 0.463 | 0.3343 | 0.2876 | - | 4 |
|  | NPRT [K-Means] | 0.463 | 0.3343 | 0.1525 | IT1 | 4 |
|  | NPRT [Cube] |  |  |  |  | 6 |
|  | NPRT [K-Means] | 0.463 | 0.3343 | 0.3968 | IT2 | 4 |
|  | NPRT [Cube] |  |  |  |  | 15 |
|  | NPRT [K-Means] | 0.463 | 0.3343 | 0.1949 | IT3 | 4 |
|  | NPRT [Cube] |  |  |  |  | 6 |
|  | NPRT [K-Means] | 0.463 | 0.3343 | 0.1416 | IT4 | 4 |
|  | NPRT [Cube] |  |  |  |  | 6 |
|  | NPRT [K-Means] | 0.463 | 0.3343 | 0.1734 | OT1 | 4 |
|  | NPRT [Cube] |  |  |  |  | 6 |
|  | NPRT [K-Means] | 0.463 | 0.3343 | 0.1951 | OT2 | 4 |
|  | NPRT [Cube] |  |  |  |  | 6 |
|  | NPRT [K-Means] | 0.463 | 0.3343 | 0.586 | OT3 | 5 |
|  | NPRT [Cube] |  |  |  |  | 15 |
|  | NPRT [K-Means] | 0.463 | 0.3343 | 0.1419 | OT4 | 4 |
|  | NPRT [Cube] |  |  |  |  | 6 |
| Incubation Park | NP | 0.9486 | 0.4094 | - | - | 3 |
|  | NPR [K-Means] | 0.9486 | 0.4094 | 0.2205 | - | 4 |
|  | NPRT [K-Means] | 0.9486 | 0.4094 | 0.0708 | IT1 | 5 |
|  | NPRT [Cube] |  |  |  |  | 7 |
|  | NPRT [K-Means] | 0.9486 | 0.4094 | 0.3474 | IT2 | 5 |
|  | NPRT [Cube] |  |  |  |  | 16 |
|  | NPRT [K-Means] | 0.9486 | 0.4094 | 0.1597 | IT3 | 4 |
|  | NPRT [Cube] |  |  |  |  | 7 |
|  | NPRT [K-Means] | 0.9486 | 0.4094 | 0.0916 | IT4 | 4 |
|  | NPRT [Cube] |  |  |  |  | 7 |
|  | NPRT [K-Means] | 0.9486 | 0.4094 | 0.1176 | OT1 | 4 |
|  | NPRT [Cube] |  |  |  |  | 7 |
|  | NPRT [K-Means] | 0.9486 | 0.4094 | 0.0982 | OT2 | 4 |
|  | NPRT [Cube] |  |  |  |  | 7 |
|  | NPRT [K-Means] | 0.9486 | 0.4094 | 0.4985 | OT3 | 5 |
|  | NPRT [Cube] |  |  |  |  | 16 |
|  | NPRT [K-Means] | 0.9486 | 0.4094 | 0.0868 | OT4 | 4 |
|  | NPRT [Cube] |  |  |  |  | 7 |

**Appendix F. Stations classification results (NP, NPR, NPRT)**

| **Station** | **Method** | **Node Value (N)** | **Place Value (P)** | **Ridership Value (R)** | **Time Span (T)** | **Class Type** |
| --- | --- | --- | --- | --- | --- | --- |
| Chengdu University of TCM & Sichuan Provincial People's Hospital | NP | 1 | 0.5588 | - | - | 3 |
|  | NPR [K-Means] | 1.0 | 0.5588 | 0.32 | - | 5 |
|  | NPRT [K-Means] | 1.0 | 0.5588 | 0.2617 | IT1 | 5 |
|  | NPRT [Cube] |  |  |  |  | 7 |
|  | NPRT [K-Means] | 1.0 | 0.5588 | 0.2169 | IT2 | 5 |
|  | NPRT [Cube] |  |  |  |  | 7 |
|  | NPRT [K-Means] | 1.0 | 0.5588 | 0.3563 | IT3 | 5 |
|  | NPRT [Cube] |  |  |  |  | 16 |
|  | NPRT [K-Means] | 1.0 | 0.5588 | 0.2198 | IT4 | 5 |
|  | NPRT [Cube] |  |  |  |  | 7 |
|  | NPRT [K-Means] | 1.0 | 0.5588 | 0.2725 | OT1 | 5 |
|  | NPRT [Cube] |  |  |  |  | 7 |
|  | NPRT [K-Means] | 1.0 | 0.5588 | 0.2246 | OT2 | 5 |
|  | NPRT [Cube] |  |  |  |  | 7 |
|  | NPRT [K-Means] | 1.0 | 0.5588 | 0.4626 | OT3 | 5 |
|  | NPRT [Cube] |  |  |  |  | 16 |
|  | NPRT [K-Means] | 1.0 | 0.5588 | 0.2036 | OT4 | 5 |
|  | NPRT [Cube] |  |  |  |  | 7 |
| Chengdu West Railway Station | NP | 0.5626 | 0.1333 | - | - | 3 |
|  | NPR [K-Means] | 0.5626 | 0.1333 | 0.2625 | - | 4 |
|  | NPRT [K-Means] | 0.5626 | 0.1333 | 0.3966 | IT1 | 4 |
|  | NPRT [Cube] |  |  |  |  | 12 |
|  | NPRT [K-Means] | 0.5626 | 0.1333 | 0.1694 | IT2 | 4 |
|  | NPRT [Cube] |  |  |  |  | 3 |
|  | NPRT [K-Means] | 0.5626 | 0.1333 | 0.2075 | IT3 | 4 |
|  | NPRT [Cube] |  |  |  |  | 3 |
|  | NPRT [K-Means] | 0.5626 | 0.1333 | 0.2192 | IT4 | 4 |
|  | NPRT [Cube] |  |  |  |  | 3 |
|  | NPRT [K-Means] | 0.5626 | 0.1333 | 0.2062 | OT1 | 4 |
|  | NPRT [Cube] |  |  |  |  | 3 |
|  | NPRT [K-Means] | 0.5626 | 0.1333 | 0.416 | OT2 | 4 |
|  | NPRT [Cube] |  |  |  |  | 12 |
|  | NPRT [K-Means] | 0.5626 | 0.1333 | 0.195 | OT3 | 4 |
|  | NPRT [Cube] |  |  |  |  | 3 |
|  | NPRT [K-Means] | 0.5626 | 0.1333 | 0.1878 | OT4 | 4 |
|  | NPRT [Cube] |  |  |  |  | 3 |
| Tongzilin | NP | 0.0667 | 0.4819 | - | - | 4 |
|  | NPR [K-Means] | 0.0667 | 0.4819 | 0.2653 | - | 3 |
|  | NPRT [K-Means] | 0.0667 | 0.4819 | 0.2447 | IT1 | 3 |
|  | NPRT [Cube] |  |  |  |  | 5 |
|  | NPRT [K-Means] | 0.0667 | 0.4819 | 0.2756 | IT2 | 3 |
|  | NPRT [Cube] |  |  |  |  | 5 |
|  | NPRT [K-Means] | 0.0667 | 0.4819 | 0.2086 | IT3 | 3 |
|  | NPRT [Cube] |  |  |  |  | 5 |
|  | NPRT [K-Means] | 0.0667 | 0.4819 | 0.1481 | IT4 | 3 |
|  | NPRT [Cube] |  |  |  |  | 5 |
|  | NPRT [K-Means] | 0.0667 | 0.4819 | 0.1887 | OT1 | 3 |
|  | NPRT [Cube] |  |  |  |  | 5 |
|  | NPRT [K-Means] | 0.0667 | 0.4819 | 0.3003 | OT2 | 3 |
|  | NPRT [Cube] |  |  |  |  | 5 |
|  | NPRT [K-Means] | 0.0667 | 0.4819 | 0.3777 | OT3 | 3 |
|  | NPRT [Cube] |  |  |  |  | 14 |
|  | NPRT [K-Means] | 0.0667 | 0.4819 | 0.1391 | OT4 | 3 |
|  | NPRT [Cube] |  |  |  |  | 5 |
| Financial City | NP | 0.0605 | 0.4265 | - | - | 4 |
|  | NPR [K-Means] | 0.0605 | 0.4265 | 0.2096 | - | 2 |
|  | NPRT [K-Means] | 0.0605 | 0.4265 | 0.054 | IT1 | 3 |
|  | NPRT [Cube] |  |  |  |  | 5 |
|  | NPRT [K-Means] | 0.0605 | 0.4265 | 0.3215 | IT2 | 3 |
|  | NPRT [Cube] |  |  |  |  | 5 |
|  | NPRT [K-Means] | 0.0605 | 0.4265 | 0.1412 | IT3 | 2 |
|  | NPRT [Cube] |  |  |  |  | 5 |
|  | NPRT [K-Means] | 0.0605 | 0.4265 | 0.0671 | IT4 | 2 |
|  | NPRT [Cube] |  |  |  |  | 5 |
|  | NPRT [K-Means] | 0.0605 | 0.4265 | 0.1212 | OT1 | 2 |
|  | NPRT [Cube] |  |  |  |  | 5 |
|  | NPRT [K-Means] | 0.0605 | 0.4265 | 0.0852 | OT2 | 3 |
|  | NPRT [Cube] |  |  |  |  | 5 |
|  | NPRT [K-Means] | 0.0605 | 0.4265 | 0.5194 | OT3 | 3 |
|  | NPRT [Cube] |  |  |  |  | 14 |
|  | NPRT [K-Means] | 0.0605 | 0.4265 | 0.0747 | OT4 | 2 |
|  | NPRT [Cube] |  |  |  |  | 5 |

**Appendix F. Stations classification results (NP, NPR, NPRT)**

| **Station** | **Method** | **Node Value (N)** | **Place Value (P)** | **Ridership Value (R)** | **Time Span (T)** | **Class Type** |
| --- | --- | --- | --- | --- | --- | --- |
| Chengdu Second People's Hospital | NP | 0.5229 | 1 | - | - | 4 |
|  | NPR [K-Means] | 0.5229 | 1.0 | 0.2677 | - | 5 |
|  | NPRT [K-Means] | 0.5229 | 1.0 | 0.2265 | IT1 | 5 |
|  | NPRT [Cube] |  |  |  |  | 9 |
|  | NPRT [K-Means] | 0.5229 | 1.0 | 0.2352 | IT2 | 5 |
|  | NPRT [Cube] |  |  |  |  | 9 |
|  | NPRT [K-Means] | 0.5229 | 1.0 | 0.2383 | IT3 | 5 |
|  | NPRT [Cube] |  |  |  |  | 9 |
|  | NPRT [K-Means] | 0.5229 | 1.0 | 0.1793 | IT4 | 5 |
|  | NPRT [Cube] |  |  |  |  | 9 |
|  | NPRT [K-Means] | 0.5229 | 1.0 | 0.2198 | OT1 | 5 |
|  | NPRT [Cube] |  |  |  |  | 9 |
|  | NPRT [K-Means] | 0.5229 | 1.0 | 0.2719 | OT2 | 5 |
|  | NPRT [Cube] |  |  |  |  | 9 |
|  | NPRT [K-Means] | 0.5229 | 1.0 | 0.3487 | OT3 | 5 |
|  | NPRT [Cube] |  |  |  |  | 18 |
|  | NPRT [K-Means] | 0.5229 | 1.0 | 0.1789 | OT4 | 5 |
|  | NPRT [Cube] |  |  |  |  | 9 |
| Hongpailou | NP | 0.1059 | 0.5064 | - | - | 4 |
|  | NPR [K-Means] | 0.1059 | 0.5064 | 0.1874 | - | 3 |
|  | NPRT [K-Means] | 0.1059 | 0.5064 | 0.217 | IT1 | 3 |
|  | NPRT [Cube] |  |  |  |  | 5 |
|  | NPRT [K-Means] | 0.1059 | 0.5064 | 0.1487 | IT2 | 3 |
|  | NPRT [Cube] |  |  |  |  | 5 |
|  | NPRT [K-Means] | 0.1059 | 0.5064 | 0.1481 | IT3 | 3 |
|  | NPRT [Cube] |  |  |  |  | 5 |
|  | NPRT [K-Means] | 0.1059 | 0.5064 | 0.1414 | IT4 | 3 |
|  | NPRT [Cube] |  |  |  |  | 5 |
|  | NPRT [K-Means] | 0.1059 | 0.5064 | 0.1466 | OT1 | 3 |
|  | NPRT [Cube] |  |  |  |  | 5 |
|  | NPRT [K-Means] | 0.1059 | 0.5064 | 0.2671 | OT2 | 3 |
|  | NPRT [Cube] |  |  |  |  | 5 |
|  | NPRT [K-Means] | 0.1059 | 0.5064 | 0.1871 | OT3 | 3 |
|  | NPRT [Cube] |  |  |  |  | 5 |
|  | NPRT [K-Means] | 0.1059 | 0.5064 | 0.1365 | OT4 | 3 |
|  | NPRT [Cube] |  |  |  |  | 5 |
| Dongmen Bridge | NP | 0.0924 | 0.6841 | - | - | 4 |
|  | NPR [K-Means] | 0.0924 | 0.6841 | 0.2171 | - | 3 |
|  | NPRT [K-Means] | 0.0924 | 0.6841 | 0.095 | IT1 | 3 |
|  | NPRT [Cube] |  |  |  |  | 8 |
|  | NPRT [K-Means] | 0.0924 | 0.6841 | 0.2743 | IT2 | 3 |
|  | NPRT [Cube] |  |  |  |  | 8 |
|  | NPRT [K-Means] | 0.0924 | 0.6841 | 0.1613 | IT3 | 3 |
|  | NPRT [Cube] |  |  |  |  | 8 |
|  | NPRT [K-Means] | 0.0924 | 0.6841 | 0.1117 | IT4 | 3 |
|  | NPRT [Cube] |  |  |  |  | 8 |
|  | NPRT [K-Means] | 0.0924 | 0.6841 | 0.1497 | OT1 | 3 |
|  | NPRT [Cube] |  |  |  |  | 8 |
|  | NPRT [K-Means] | 0.0924 | 0.6841 | 0.1466 | OT2 | 3 |
|  | NPRT [Cube] |  |  |  |  | 8 |
|  | NPRT [K-Means] | 0.0924 | 0.6841 | 0.4308 | OT3 | 3 |
|  | NPRT [Cube] |  |  |  |  | 17 |
|  | NPRT [K-Means] | 0.0924 | 0.6841 | 0.1179 | OT4 | 3 |
|  | NPRT [Cube] |  |  |  |  | 8 |
| Huaxiba | NP | 0.0776 | 0.6228 | - | - | 4 |
|  | NPR [K-Means] | 0.0776 | 0.6228 | 0.2433 | - | 3 |
|  | NPRT [K-Means] | 0.0776 | 0.6228 | 0.157 | IT1 | 3 |
|  | NPRT [Cube] |  |  |  |  | 5 |
|  | NPRT [K-Means] | 0.0776 | 0.6228 | 0.1545 | IT2 | 3 |
|  | NPRT [Cube] |  |  |  |  | 5 |
|  | NPRT [K-Means] | 0.0776 | 0.6228 | 0.3082 | IT3 | 3 |
|  | NPRT [Cube] |  |  |  |  | 5 |
|  | NPRT [K-Means] | 0.0776 | 0.6228 | 0.119 | IT4 | 3 |
|  | NPRT [Cube] |  |  |  |  | 5 |
|  | NPRT [K-Means] | 0.0776 | 0.6228 | 0.2171 | OT1 | 3 |
|  | NPRT [Cube] |  |  |  |  | 5 |
|  | NPRT [K-Means] | 0.0776 | 0.6228 | 0.1205 | OT2 | 3 |
|  | NPRT [Cube] |  |  |  |  | 5 |
|  | NPRT [K-Means] | 0.0776 | 0.6228 | 0.4128 | OT3 | 3 |
|  | NPRT [Cube] |  |  |  |  | 14 |
|  | NPRT [K-Means] | 0.0776 | 0.6228 | 0.1126 | OT4 | 3 |
|  | NPRT [Cube] |  |  |  |  | 5 |
